# Small is beautiful? Explaining resident satisfaction in Swedish nursing home care

**DOI:** 10.1186/s12913-019-4694-9

**Published:** 2019-11-25

**Authors:** Douglas Spangler, Paula Blomqvist, Ylva Lindberg, Ulrika Winblad

**Affiliations:** 1 Department of Public Health and Caring Sciences, Uppsala University, Box 564, 751 22 Uppsala, Sweden; 2 Department of Government, Uppsala University, Box 514, 751 20 Uppsala, Sweden; 3 Department of Public Health and Caring Sciences, Uppsala University, Box 564, 751 22 Uppsala, Sweden; 4 Department of Public Health and Caring Sciences, Uppsala University, Box 564, 751 22 Uppsala, Sweden

**Keywords:** Nursing home, Eldercare, Quality, Satisfaction, Sweden

## Abstract

**Background:**

Resident satisfaction is an important aspect of nursing home quality. Despite this, few studies have systematically investigated what aspects of nursing home care are most strongly associated with satisfaction. In Sweden, a large number of processual and structural measures are collected to describe the quality of nursing home care, though the impact of these measures on outcomes including resident satisfaction is poorly understood.

**Methods:**

A cross-sectional analysis of data collected in two nationally representative surveys of Swedish eldercare quality using multi-level models to account for geographic differences.

**Results:**

Of the factors examined, nursing home size was found to be the most important predictor of resident satisfaction, followed by the amount of exercise and activities offered by the nursing home. Measures of individualized care processes, ownership status, staffing ratios, and staff education levels were also weakly associated with resident satisfaction. Contrary to previous research, we found no clear differences between processual and structural variables in terms of their association with resident satisfaction.

**Conclusions:**

The results suggest that of the investigated aspects of nursing home care, the size of the nursing home and the amount activities offered to residents were the strongest predictors of satisfaction. Investigation of the mechanisms behind the higher levels of satisfaction found at smaller nursing homes may be a fruitful avenue for further research.

## Background

The increasingly elderly population in many western countries has created an increased demand for high quality medical and social care services. This includes nursing home (NH) care, referring to facilities providing 24-h functional support and care for persons who require assistance with activities of daily living and who often have complex healthcare needs [1]. Achieving quality in NH care is complicated by the fact that care quality is multifaceted, difficult to define and measure, and may be perceived differently by different stakeholders [2]. Regulatory agencies thus often struggle to identify factors most important in achieving high-quality NH care [3].

A particular challenge in regulating quality in NH care is that it is in many regards a ‘soft’ service in which the individual experiences of the NH residents is an important dimension of quality. While many aspects of quality (e.g, clinical quality and cost effectiveness) must be considered in order to achieve a well-rounded assessment of the care provided at a given nursing home, some scholars have argued that resident satisfaction may be the most appropriate assessment of quality in NH care [4, 5]. In health care, investigations of patient satisfaction are abundant [6, 7], while studies measuring NH resident satisfaction are less common. This may be due to the suggestion that elderly patients with cognitive weaknesses have difficulty reliably answering surveys [5], though studies have shown that patients in cognitive decline are capable of answering surveys, particularly if they are designed with their needs in mind [8–11].

Given that the satisfaction of residents is an important dimension of quality in NH care, the question becomes how this is achieved. That is to say, what factors are most important to focus on when seeking to improve the satisfaction of NH residents? The most commonly used analytical framework for understanding how quality is generated in health and social care is Donabedian’s *structure – process – outcome* model [12, 13]. A central distinction in Donabedian’s model is that between structural and processual quality factors, which are seen as potential explanatory factors behind quality outcomes. *Structural* factors refer to the physical attributes of the setting in which care is provided, including the number and qualifications of staff, equipment, and physical facilities [13]. *Processual* factors denote the manner in which the care services are delivered, e.g. whether care routines follow set guidelines, and the extent to which residents are involved in decisions about their care. Quality *outcomes* can be measured in many ways, both objectively in the form of health status or subjectively in the form of patient/resident satisfaction [12]. A central unresolved question posed in Donabedian’s work is whether structural or processual measures are most important for generating outcome quality, and precisely how these factors interact to produce the desired outcomes.

The literature on medical quality in NH care in terms of, for instance, mortality and adverse event rates, has investigated numerous explanatory factors including staffing, ownership, care routines, and the size of facilities [14–17]. Such studies are particularly abundant in the United States, where collection of the Minimum Data Set provides a robust basis for performing broad studies of clinical outcomes. There are considerably fewer investigations of the determinants of resident satisfaction. Previous studies have investigated structural factors including staff satisfaction [18], and job commitment [19], with both studies finding positive associations with resident satisfaction. A broader study of the influence of organizational factors found that NH ownership, staffing levels, and the provision of family councils were important predictors of NH resident satisfaction [20]. Others have investigated specific interventions related to processual quality factors such as improved meal time routines [21], “person-centered care” initiatives [22], and social activity programs such as gardening [23]. While generally finding positive effects on resident satisfaction, these interventional studies are narrow, and differ in terms of setting and methodology, making them difficult to compare. Taken together, the prior literature on what factors are associated with resident satisfaction in NHs is largely limited to evaluations of specific interventions, and there are few studies investigating the relative influence of structural and processual factors, particularly in the European context.

In Sweden, several public investigations have pointed to quality deficiencies, and a lack of systematic knowledge about factors leading to improved quality [24, 25]. The issue of NH care quality has increased in significance in Swedish public debate as reforms have led to an increasing number of homes contracted out by local governments (municipalities) to private, often for-profit firms. In 2017, one study found that about one fifth of the Swedish NHs were run by for-profit providers [26]. This study, as well as another recent investigation of Danish NHs, found that overall, privately operated homes outperformed public and non-profit homes in terms of process measures, while underperforming in terms of structural measures [26, 27]. Neither of these studies investigated resident satisfaction however.

In Sweden, there is good availability of data on various aspects of NH care due to comprehensive data collection efforts by the Swedish National Board of Health and Welfare (NBHW). Annual surveys measuring satisfaction are sent by the NBHW to all NH residents, and surveys assessing processual and structural measures of quality are sent to every NH in Sweden. So far however, the use of these data for research has been limited. One exception is a study by Kajonius and Kazemi [28] which investigated differences in satisfaction among NH residents at the municipal level, finding that processual quality factors such as respect and access to information appeared to be more important for residents than structural factors such as staffing and budget.

In this study, we aim to evaluate which structural and processual measures of quality have the strongest associations with overall NH resident satisfaction. In doing so, we hope to provide policymakers and researchers with a broader picture of the determinants of resident satisfaction as NHs than has previously been available.

## Methods

### Setting

In Sweden, all citizens have access to publicly funded NH services at heavily subsidized rates. The eldercare system in Sweden is decentralized, with responsibility for service provision resting with the nation’s 290 municipalities. Municipalities are obliged to offer NH care to those determined to have a need for such care based on national criteria. The municipality may provide services themselves, or contract out service provision to private entities [29]. In 2016, there were in total 88,886 individuals [30] living in ca. 2300 NHs in Sweden [31], with 20.5% of residents living in NHs operated by private providers [30]. While marketization reforms have led to an increase in the proportion of privately managed NHs, they remain publicly funded [32]. All NHs, both public and private, are subjected to the same national quality reporting requirements, user safety regulations, and auditing measures [33]. This study includes all NHs in Sweden providing care to individuals over 65 years of age in 2016, excluding facilities offering only short-term care.

### Data collection

Two nationally representative surveys conducted in 2016, both developed and administered by the NBHW, serve as the primary sources of data. The first survey is a user satisfaction survey (*Brukarundersökningen,* or user survey) distributed yearly to all individuals over 65 years of age receiving elder care services including NH care. This survey consists of 27 separate items to be rated on a five-point Likert scale, relating to their satisfaction with a variety of aspects of elder care services, as well as their health status. Among those living in NHs the survey had a response rate of 56% in 2016, resulting in a total of 40,371 responses [34].

The second data source is a survey sent directly to all NHs in Sweden by the NBHW, which assesses a number of processual and structural measures of quality. This survey (*Enhetsundersökningen*, or unit survey) is completed by administrative staff at each NH, and had a response rate of 93% in 2016, resulting in 2153 responses [35]. In addition to quality measures, the unit survey provides data on the type of services provided by the NH (general, dementia and/or assisted living), the number of residents in each home, and whether the NH is operated by a public or private entity. While the NBHW has a long experience of developing and administering surveys, and assessments of loss-to follow-up in the user survey have been performed [36] the psychometric properties of these surveys have not been published in the publicly available literature .

Observations in the two NBHW survey datasets for 2016 were matched based on the NH name and municipality. This involved both an automated matching process, and a subsequent manual review of unmatched records. Municipality-level variables were extracted from the national municipality and county council database Kolada [37] and merged into the dataset.

### Variables

Variables for analysis were aggregated from the two surveys based on their conceptual meaning and the results of an exploratory factor analysis which may be found in Additional file 1, p 1–7. The extracted variables are detailed below, and a summary of the categorization is available as Additional file 2.

### Dependent variable

Upon exploratory factor analysis, it was found that questions in the user survey were highly correlated (Cronbach’s Alpha = 0.92), and was a poor candidate for approaches based on extraction of distinct latent variables. As such, we chose to extract a single composite measure of satisfaction from the user survey for use as the dependent variable, consisting of questions 5–19, 21–25, and 27. To generate a composite measure for use as the dependent variable, the percent of residents at a nursing home responding positively to a given survey question was normalized by subtracting the average percentage of patients responding positively to that question in the population, and dividing by the standard deviation of the population, resulting in a standardized z-score. Z-scores were then averaged across all included survey items to result in a composite score with equal weights for each question.

### Independent variables

The NBHW divided the unit survey into 12 conceptual categories. A factor analysis showed that the individual questions generally loaded well onto the categories proposed by the NBHW and it was therefore chosen, with a few exceptions, to retain this categorization as the basis for the independent variables used in the analysis. Based on the Donabedian model, the independent variables were divided into “structural” and “processual” variables.

### Processual variables

The first seven variables related to different processual factors, such as meal-related routines or physical or social activities.

Questions 1 and 1a in the unit survey related to the ability of residents to participate in “resident councils” where residents regularly meet to voice concerns in the NH. Issues raised during resident councils may for instance include the planning of common activities or menus for the coming weeks. These were aggregated and reported as the variable *Participation in resident councils.*

Questions 2 and 3 in the unit survey concerned the existence of-, and the residents participation in, the creation of “action plans” concerning the care needs and wishes of the resident. These action plans contain information about how various care activities are to be carried out and should be updated every 6 months. The questions were combined into the variable *Individualized action plans*.

Questions 4 and 5 addressed the existence of meal-related routines, and the documentation of meal preferences in the residents’ action plans. Such meal routines are to be based on the Five Aspects Meal Model (FAMM) proposed by Gustafsson et al. [38], and should be updated every 24 months. The questions were combined into the variable *Meal-related routines and plans*.

Questions 6a-c in the survey related to the existence of formal routines for handling resident safety issues such as threats, violence, and addiction. While the NBHW grouped question 7 (routines for cooperation with relatives) into this category, it did not load well onto a common factor and is conceptually quite distinct, and was therefore excluded. The remaining questions were combined into the variable *Patient safety routines*.

Questions 8 and 8a-b in the unit survey related to facilities for-, and availability of, exercise and social activities. We excluded question 8 (whether the NH residents have access to facilities for physical activity), which had a weak-to-moderate factor loading, so as to interpret this variable as a purely process-related measure. The remaining questions were combined into the variable *Availability of exercise and social activity*.

Questions 9 and 10 related to the existence of routines for planning care in cooperation with other healthcare providers, and whether resident’s involvement was documented. Similarly, questions 11 and 12 related to routines for medication reviews and whether resident participation is documented in the medical record. We reported these as the variables *Care coordination routines* and *Medication review routines*, respectively.

### Structural variables

The structural variables included indicators of staffing, ownership and size. Three factors relating to staffing from the unit survey, including the ratio of nurses per resident (questions 13 and 14), non-nurse staff per resident (questions 15 and 16), and the portion of staff with an “adequate education” for their position (questions 17 & 18) were identified. These are reported as the variables *Nurses per resident, Staff per resident,* and *Staff with adequate education* respectively, and weekday and weekend staffing levels were weighted at a 5:2 ratio to represent average daily staffing levels. While staffing ratios are fairly straightforward to calculate, the definition of what constitutes an “adequate education” is more complex. Adequacy is determined by the amount of healthcare-related training completed by non-nurse staff based on a point scale established by the NBHW [39].

The number of beds available at each NH was reported as *Size of nursing home*. The NH’s ownership status, i.e. whether it was run by a private or a public provider, was reported as the variable *Private ownership.*

### Controls

Several variables were included in the analysis to control for population health differences between the NHs included in this study. Self-rated health has been found to be an excellent predictor of clinical outcomes [40, 41], and we used questions 1–3 and 20 in the user satisfaction survey, which asked about the residents’ physical and mental well-being, to control for health status. The type of facilities (general, dementia and/or assisted living) available at the NH was also controlled for.

It was further deemed necessary to control for demographic factors for which data was only available at the municipal level. This refers to different demographic, economical, and political conditions which may vary significantly between the 290 municipalities. A set of controls were adapted from previous studies [26, 42, 43] including per capita income levels, population density, age profiles, political control, and expenditures, the details of which may be found in Table 1. Data at the municipality level was collected from the Kolada database [37].

**Table 1:** Descriptive statistics of aggregate variables

	Mean	SD	Median	IQR	Missing
Dependent variable					
Aggregate resident satisfaction	0.01*	1.00*	0.05	1.34	4
Processual variables					
Participation in resident councils	0.00*	1.00*	0.48	1.63	0
Individualized action plans	0.00*	1.00*	0.48	1.00	0
Meal-related routines and plans	0.00*	1.00*	−0.17	1.60	53
Patient safety routines	0.00*	1.00*	−0.35	1.98	0
Care coordination routines	0.00*	1.00*	−0.05	2.07	0
Medication review routines	0.00*	1.00*	0.09	2.18	0
Availability of exercise and activities	0.00*	1.00*	0.08	1.82	0
Structural variables					
Private ownership of nursing home	0.19	0.39	0.00	0.00	1
Size of nursing home	43.57	22.70	39.00	25.00	6
Nurses per resident	0.03	0.01	0.03	0.02	62
Staff per resident	0.29	0.06	0.28	0.06	41
Staff with adequate education	83.71	14.12	86.86	18.54	40
Has general care facilities	0.79	0.41	1.00	0.00	0
Has dementia care facilities	0.59	0.49	1.00	1.00	0
Has assisted living facilities	0.05	0.23	0.00	0.00	0
Resident Health Controls					
Aggregate self-rated health	0.01*	1.00*	−0.04	1.31	12
Municipal controls (Weighted by # of nursing homes in Municipality)					
Population 65+ in Nursing Home (%)	4.21	0.88	4.21	0.99	19
Population 65+ (%)	21.22	4.19	21.20	6.33	0
Population per square kilometer	472.4	1164.7	60.6	116	0
Average annual cost per resident (SEK)	838,285	161,812	822,686	117,267	19
Average age of residents in nursing homes	83.49	1.82	83.60	2.30	0
Political control (left = −1, mixed = 0, right = 1)	−0.12	0.80	0.00	2.00	0
Average annual per capita taxable income (SEK)	188,232	24,921	183,269	23,691	0

*These variables are mean centered and normalized. The reader may draw conclusions regarding the distribution of the normalized variables by examining the median to determine skew, and IQR to assess for kurtosis (a standard normal distribution has an IQR of 1.35)

### Statistical analysis

As the large number of quality measures made available by the NBHW was unsuited to direct inclusion in a regression-modelling framework, an initial exploratory factor analysis was performed to reduce the dimensionality of the dataset as described above. Data from the user satisfaction survey and the unit survey were aggregated at the NH level. We sought to minimize bias in the estimation of the effects of the investigated quality measures by drawing upon the approach to causal modelling first described by Pearl [44], using the assumptions of causal directionality described by the Donabedian model of healthcare quality [12, 13]. The Donabedian model asserts that a causal relationship exists between structural and processual aspects of healthcare quality, and we assumed that the satisfaction of NH residents would be confounded by their health status. To control for confounding due to these causal relationships, the effects of processual measures of quality were modeled controlling for resident health and structural measures of quality. We present coefficient estimates for structural measures including controls for other measures of structural quality, though the direction of causality within the selected set of structural measures is in many cases unclear. In addition to these full models, we present additional nested models estimating bivariate associations, and models controlling only for resident health. In this framework, variations in the regression coefficients between the full and nested models allowed for the interpretation of the impact of health status and structural factors on the effect of the quality measures.

The aggregated variables were first analyzed in a classical ordinary least squares regression framework using the Huber-White sandwich estimator to account for heteroscedasticity and clustering as implemented in the *rms* R package [45]. Hierarchal models including municipality-level controls with random intercepts for municipalities were implemented using a “Partial pooling” approach to account for clustering and confounding due to municipal-level factors [46], as implemented in the *lme4* R package [47]. Confidence intervals were generated using basic parametric bootstrap resampling.

In this analysis, we report our results in terms of standardized regression coefficients. While this allows for direct comparison of the importance of each independent variable in predicting resident satisfaction, it makes interpretation in terms of absolute effects cumbersome. Given the low rates of missing data at the unit level, multiple imputation was not deemed to be necessary, and cases with missing values were deleted list-wise in the relevant models. All statistical analyses were performed using R version 3.5.0, and a reproducible accounting of our reported findings is included as Additional file 1. A number of sensitivity analyses investigating the impact of various model specifications, potential biases due to loss to follow-up, and assumptions made in the main analysis are also included in Additional file 1. Source code and the data necessary to reproduce these findings are available on Mendeley Data [48].

## Results

Data from both surveys (the user survey and the unit survey) were aggregated at the NH level, resulting in 1921 records in the user survey, and 2189 records in the unit survey. 1711 records could be automatically linked based on municipality and NH names, and an additional 87 records could be matched through manual review, resulting in a dataset containing 1798 NHs. An analysis of non-matched records may be found in Additional file 1. p 7–8. An analysis of the association between survey response rates and the investigated variables was performed. We found a positive association between response rates and resident satisfaction, as well as a negative association between response rates and nursing home size, and an effect indicating that private nursing homes had higher response rates (See dropout analysis in Additional file 1, p 8). Generally, residents of NHs were quite satisfied; in the 2016 survey, 83% answered that they overall were fairly or very satisfied with the care they received.

### Descriptive data

Descriptive statistics were generated for each of the variables included in the analysis, and are presented in Table 1. We found that the average NH in Sweden has space for 43 residents, a resident to staff ratio of roughly 3.5:1, a resident to nurse ratio of 30:1, and that 83% of non-nurse staff had an adequate level of education as defined by the NBHW criteria. 19% of included NHs were operated by private providers. 80% of NHs offered general care services, while 60% offered dementia care services, and only 5% had assisted living facilities – These sum up to over 100% as a single NH can offer more than one type of service.

With regard to municipality level statistics, we see that about 21% of Swedes are over the age of 65, 4% of whom live in NHs, where the average age of residents is 83. The average annual per-resident cost for the municipality is 838 thousand SEK (around 80 thousand EUR), while average per capita taxable income is 188 thousand SEK (Table 1).

### Regression analysis

**Fig. 1:**
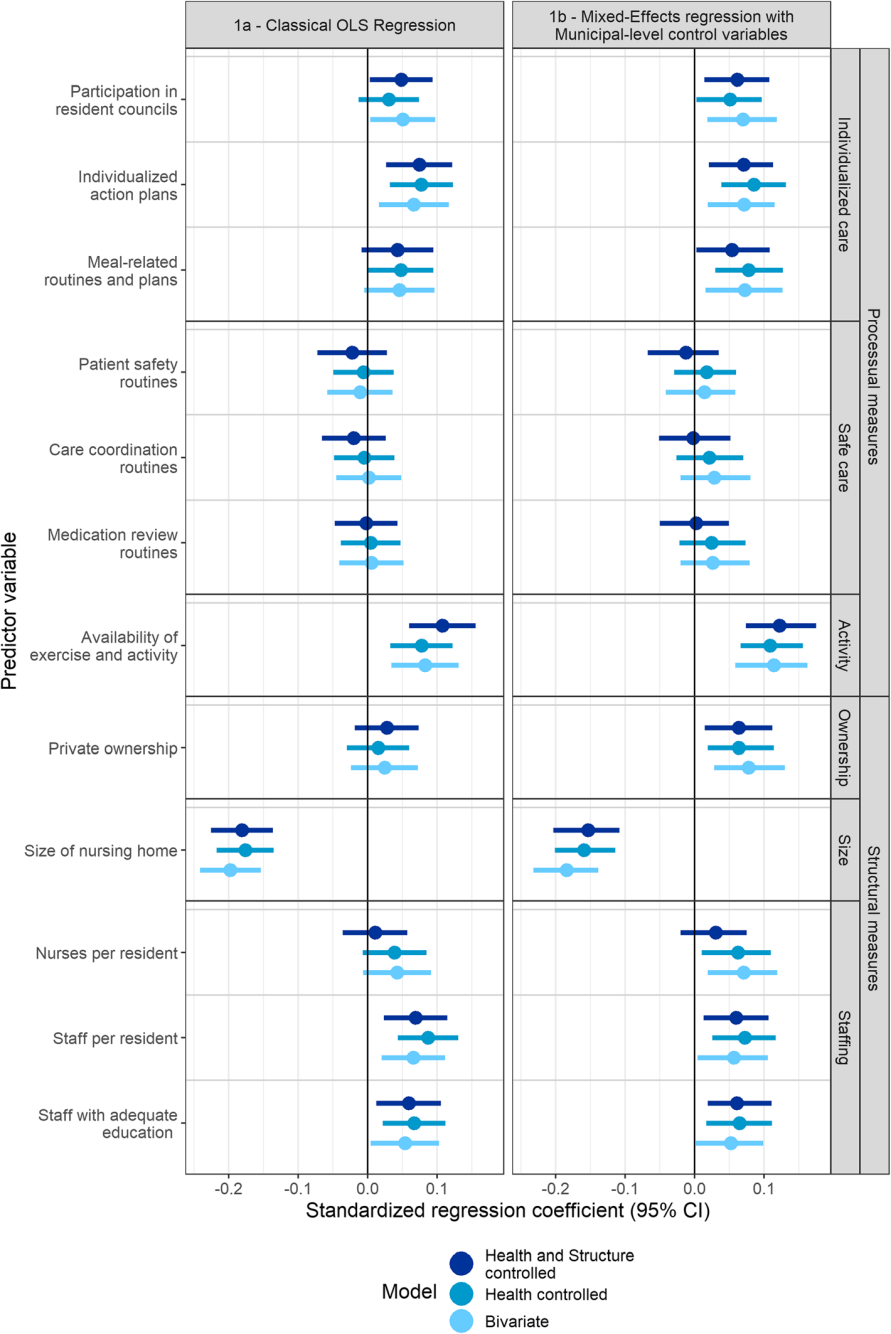
Standardized regression coefficients of predictors for composite resident satisfaction. This figure presents standardized regression coefficients for each of the analyzed independent variables. Coefficients are presented for models including only the relevant independent variable (bivariate), with controls for only the health status of the patient (Health controlled), and with controls for both health status and the structural measures (Health and Structure controlled)

Figure 1 presents the summarized results of each of the models developed to characterize the independent variables created from the unit survey. 1a presents the results using a classical OLS regression framework, while 1b presents the results of hierarchal mixed-effects models controlling for municipal level effects.

In terms of overall predictive value, an OLS model including all covariates achieved an adjusted r^2^ of 0.182, while the conditional r^2^ value [49] of the multi-level model containing all predictor variables was 0.254. In the multi-level framework, we found that variation between municipalities accounted for 10% of the total variation found between NHs. A total of 12 processual and structural variables were extracted from the unit survey for analysis as independent variables. Upon analyzing the results, variable groupings were identified post hoc based on similarities with regard to effect sizes and conceptual meanings, which are used to simplify the discussion of our findings, and are labelled on the right-hand side of Fig. 1.

The variables in the first group, labelled *Individualized care*, are all related to the individual care process. They include the variables *Participation in resident councils*, *Individualized care plans*, and *Meal-related routines and plans.* This group had an average effect size of 0.06 in our fully controlled models, and while 95% confidence intervals in the main model consistently excluded zero after adjusting for municipality-level covariates. The significance of the variables in this group varied upon sensitivity analyses however (See Additional file 1, p 22–25).

The next group, labelled *Safe care*, includes the variables *Patient safety routines*, *Care coordination routines*, and *Medication review routines*. They are all related to the existence of formal guidelines dealing with various aspects of care. As seen in Fig. 1, none of these variables displayed significant correlations to resident satisfaction.

The final group in the processual category consists of only one variable, *Availability of exercise and social activity*. This variable, labelled *Activity*, displayed the highest degree of correlation with overall resident satisfaction among the process variables, with an effect size of 0.11 in our fully controlled model, and was robust across a range of sensitivity analyses.

Turning to the structural variables, another three variable groups were identified. We identified no significant effects in the OLS model with regard to ownership status. Upon controlling for municipality-level variables, a significant positive correlation with a magnitude of 0.06 in the fully controlled model was found, though the significance of the association was sensitive to variations in model specifications.

The *Size* of the NH was by a significant margin the most important predictor of resident satisfaction in this analysis, with the negative coefficient suggesting that smaller NHs are associated with more satisfied residents. A small decrease in the effect of this variable could be noticed upon controlling for municipality level effects, suggesting that larger NHs may be more common in municipalities where residents are on average, less satisfied with their NH care. The effect of size was robust in our sensitivity analyses.

The third group of structural variables included *Nurses per resident*, *Staff per resident* and *Staff with adequate education*, and was labelled *Staffing*. The group as a whole had an average effect size of 0.05 among the fully controlled models. With the exception of nurse staffing ratios, 95% confidence intervals consistently excluded zero in the main models, but the significance of the effect was sensitive to varying model specifications.

Taken together, the results of the analysis presented in Fig. 1 show that the structural measure *Size* of the NH was the most important predictor of resident satisfaction, followed by the processual *Availability of exercise and social activity* variable. The effects of the processual *Individualized care* variables and the structural *Staffing* variables were similar in magnitude, as was the effect of *Private ownership,* upon controlling for municipality-level effects. These effects were also sensitive to alternate model specifications. The processual *Safe care* variables were not found to have any significant association with resident satisfaction.

Finally, a comment on the significant effects found among our control variables is in order. In our fully controlled model, self-rated health was found to have a strong positive correlation with satisfaction (standardized regression coefficient of 0.34) suggesting that healthier residents reported considerably higher levels of satisfaction. Among the municipality level controls, average NH resident age had a positive correlation with satisfaction, and average per capita taxable income had a negative correlation with satisfaction. Interestingly, no significant relationship between the amount spent per resident and satisfaction was identified. Full model summaries, along with a table reporting the data upon which Fig. 1 is based may be found in Additional file 1, p 12–15.

## Discussion

In this study, we investigated a total of 12 variables representing different aspects of care quality reported in the NBHW unit survey. Of these, seven were considered to represent process-related quality, and five to represent structural quality. Our main findings were that the *Size* of a NH (a structural measure) had the greatest impact on resident satisfaction, followed by the processual measure A*vailability of exercise and social activities*. The processual variables concerning *Individualized care* and the structural *Staffing* and *Private ownership* all had similar, weakly positive, effects on resident satisfaction. The processual *Safe care* variables had no significant effect on resident satisfaction. We found no clear differences in terms of effect sizes between processual and structural variables. Below, we discuss these findings in order of the effect size identified in our results.

The fact that NH *size* was the best predictor of resident satisfaction suggests that smaller NHs in Sweden had more satisfied residents than their larger counterparts. A recent literature review surveying studies examining the impact of NH size on quality outcomes showed size to be an important predictor of quality, with smaller homes generally having better quality outcomes [15]. None of the 30 studies investigated the relationship between size and resident satisfaction, though five investigated similar composite “Quality of Life” measures. There are however some indications that larger nursing homes may be associated with better clinical outcomes such as lower hospitalization risks [50] and lower rates of antipsychotic medication use [51]. NH quality is a multi-faceted concept, and it is not necessarily the case that the determinants of quality will affect all aspects of quality in the same way. As such, while this study does add to the evidence that smaller NHs are associated with the type of “soft” quality which resident satisfaction may be said to represent, the results should not be interpreted as saying anything regarding “harder” measures including clinical outcomes, the determinants of which may be quite different.

While size may be an important predictor of satisfaction in and of itself, it is also likely that there are causal mechanisms behind this association which mediate the effect of size. Previous research has for instance indicated that staff turnover may be lower [52] and staff continuity higher [53] at smaller NHs. The findings of this study thus emphasize the importance of identifying the more proximal mechanisms by which smaller NHs generate higher levels of satisfaction. The interpersonal aspects of nursing home care which these measures reflect are however difficult to measure, and investigating the mechanisms behind these softer dimensions of nursing home care may require a more qualitative approach.

The A*vailability of exercise and social activities* was found to have the strongest association with resident satisfaction among the processual variables. Previous research has found that physical activity-related interventions can improve the subjective health status of NH residents [54], although other studies have found weaker or even negative effects [55]. Our results suggest that, overall, NHs which offer more frequent opportunities for exercise and social activity have higher levels of resident satisfaction. The effect of activity was not diminished by controlling for resident health or NH structure; rather, the effect increased slightly suggesting that the provision of such activities may be even more important at NHs with poorer structural preconditions, particularly with regard to facility size.

Three other variable groups had weaker effects with regards to resident satisfaction: *Individualized care, Private ownership,* and *Staffing.* The *Individualized care* variables included participation in resident councils, the use of individualized care plans and the use of meal routines. We identified no previous research regarding the impact of resident councils or the use of individualized care plans on satisfaction in the literature, though Lucas et al., [20] did identify a positive impact of similar “family councils”. Our findings suggest that these quality improvement measures may indeed be associated with higher levels of resident satisfaction, although more directed studies are necessary to confirm this. There is some evidence that interventions to improve meal-related processes are effective [56, 57], and our results are consistent with a positive impact of such improvements on resident satisfaction.

The structural measures related to *staffing* had effect sizes similar to those found among the processual *individualized care* measures. Staffing as a determinant of care quality has been well researched. In a review of 70 articles, Castle [58] found a preponderance of evidence suggesting that increased staffing levels are positively associated with several measures of NH care quality. More recent studies by Castle and Anderson [59], Hyer et al. [60], and Shin and Hyun [61] point to similar results. However, none of these studies investigated effects on resident satisfaction. We found that both non-nurse staffing ratios and education levels were associated with resident satisfaction in all models, while nurse to resident ratios were significant upon controlling for municipal-level factors, and effect sizes were reduced upon controlling for other structural factors. Our results are thus consistent with a positive relationship between staffing levels and NH care quality.

Regarding the effect of ownership, the main results suggest a higher level of resident satisfaction among privately operated NHs after controlling for municipal level covariates. That is to say, while there was no overall difference in absolute levels of satisfaction, a difference was identified upon taking into account that public and private NHs are not evenly distributed across Sweden, and that when the effects of this non-uniform distribution was accounted for (in effect comparing NHs within the same county), a difference could be identified. The somewhat counter-intuitive effect could, at least in part, be explained by the tendency of private care providers in Sweden to establish themselves in municipalities with higher income levels, where resident expectations may be higher. This supposition is supported by the finding that average per capita income had a significant negative association with resident satisfaction (see Additional file 1, p 17). The significance of ownership status was not robust in sensitivity analyses however, and as such constitutes quite weak evidence for the superiority of private over public nursing homes with regards to resident satisfaction.

While we found no association between measures of *safe care* and resident satisfaction, it stands to reason that the processes which these measures represent (e.g. the performance of regular medication reviews and the existence of care coordination plans) are not immediately visible to residents, and are thus less likely to influence satisfaction. Studies investigating the impact of these measures on clinical outcomes may well find that they do have an effect with regards to quality in that respect.

Taken together, the findings of this study indicate that NH residents are more satisfied in smaller NHs, and NHs with frequent opportunities for physical and social activity. Only weak effects were identified with regards to processual individualized care measures, private nursing home ownership, and staffing levels. Formal routines had no effect on the satisfaction of residents. Another contribution of the study is the comparison of the effect of structural and processual variables on satisfaction. In contrast to a previous study on Swedish NH care [28], this study did not lend support to any firm conclusions regarding the superiority of one type of quality measure over the other. Rather, it was demonstrated that both structural variables such as size, staffing and ownership, *and* processual variables including individualized care and activities play a role in determining resident satisfaction. The difference in results between the two studies could be explained by the fact that the processual and outcome variables in the Kajonius and Kazemi study were both drawn from the resident survey (which we found upon factor analysis to be highly inter-correlated), while the structural variables they were compared with were drawn from a separate statistical database lacking this overall correlation. It is thus likely that the differential effects identified by Kajonius and Kazemi are an artefact of how the authors chose to operationalize the processual and structural measures. Furthermore, in the study data was aggregated at the municipal level, thereby investigating only differences in resident satisfaction between municipalities, which we found to account for only 10% of the total variation in satisfaction between NHs.

### Strengths and limitations

This study was a secondary analysis of two nationally representative surveys collected for quality improvement purposes. A strength of the study is thus that the results are likely to generalize well to other contexts similar to that of Sweden, and the wide scope of these surveys allowed us to investigate and compare a broad range of factors. A limitation of the study was that the validity and reliability of these surveys has not been established in the publicly available literature, although the NHBW has analyzed the impact of loss to follow-up in the user survey [62], and performs ongoing internal quality assurance of the surveys it conducts. Another risk involved in the secondary analysis of data is the proliferation of “researcher degrees of freedom” arising from the numerous decisions which must be made in transforming and analyzing such data [63]. To ameliorate these risks, we sought to define our analysis strategy a priori, and provide the resources necessary to fully reproduce our results [48]. Another limitation is that the aggregate data used in this study precludes the interpretation results in terms of individual-level effects, and readers must be careful to not commit the “ecological fallacy” of interpreting effects operative at the NH level as applying to individuals.

Among other simplifying statistical assumptions including those of additivity and linear effects, we assumed that each question in the survey was equally important to residents in generating the composite measure used as the dependent in our analysis. Weighting each question equally would seem to be a reasonable assumption to make in the absence evidence regarding resident preferences, and the main findings regarding nursing home size and availability of activities were robust to a range of sensitivity analyses and alternate survey question weights.

It was common for the satisfaction surveys to be completed with the assistance of third parties, which could potentially influence reported outcomes, and while the rate of missing data was too high to include this variable in the formal analysis, a sub group analysis of homes reporting data on this variable may be found in Additional file 1, p 21–22. Based on our findings, we do not expect this factor to be a threat to the validity of our results. We also analyzed the associations present within the user survey data between NH level response rates and the quality measurements reported in the study. We identified a positive correlation between response rates and satisfaction rates, as has been found in previous studies of this phenomenon [64, 65]. We also identified effects suggesting that response rates were higher at smaller nursing homes, and at private nursing homes (See Additional file 1, p 8). Previous studies have suggested that low response rates are likely to result in an over-estimation of satisfaction [64]. As such, bias resulting from the systematic differences in response rates would likely be in the direction of under-estimating the association of size and private ownership with satisfaction.

## Conclusions

Of the quality factors investigated, NH size had the most prominent association with satisfaction, followed by the availability of exercise and social activities. Processual measures relating to individualized care, such as participation in resident councils and the formulation of individualized action plans had a weak association with resident satisfaction, as did other structural factors such as staffing ratios and staff education. The results also suggested that privately managed NHs had a slightly higher level of resident satisfaction, though the effect was similarly weak and appeared only after adjusting for municipality-level covariates. The results in this study suggest that both structural and processual quality factors matter in determining resident satisfaction, with NH size and the availability of exercise and activities having the greatest impact.

### Implications for policy and practice

While the findings in this study suggest a direct link between offering more activities and a higher rate of satisfaction, more research is needed to determine why residents appear more satisfied at smaller homes. It may be that the proximal causes of satisfaction at smaller NHs could be replicated at their larger counterparts, for instance by improving staff continuity and turnover. If so, this could be a cost-effective alternative to building smaller nursing homes. Qualitative studies using methods such as interviews and participant observation may be most appropriate to investigate such effects in more depth. Another policy implication is that activities for residents should be a priority in NH care, and in cases where NHs care is contracted out, offering physical and social activities should be a requirement.

## Supplementary information

**Additional file 1.** Analysis_notebook. This document provides additional details regarding the factor analysis undertaken to reduce the dimensionality of the data prior to regression analysis, additional details regarding the main analysis, and a number of post-hoc analyses undertaken to evaluate the sensitivity of the findings, and investigate a number of interesting findings suitable for pursuit in further research.

**Additional file 2.** Survey_questions. This document details the specific questions from the two NHBW surveys constituting the aggregate variables included as independent variables in the regression analysis reported in this manuscript.

## Data Availability

All data used in this study are publicly available. The data and code use to generate these results are available on Mendeley data at: 10.17632/y69zhgxym3.2

## Abbreviations

FAMM: Five Aspects Meal Model
IQR: Inter-Quartile Range
NBHW: National Board of Health and Welfare
NH: Nursing Home
OLS: Ordinary Least Squares regression
SEK: Swedish Krona
