# Supplementary material for: Small is beautiful? Explaining resident satisfaction in Swedish nursing home care
Source: BMC Health Serv Res. 2019 Nov 25;19:886. doi: 10.1186/s12913-019-4694-9 (PMC6878673; doi:10.1186/s12913-019-4694-9)
Supplement: Supplementary file 1 — Additional file 1. Analysis_notebook. This document provides additional details regarding the factor analysis undertaken to reduce the dimensionality of the data prior to regression analysis, additional details regarding the main analysis, and a number of post-hoc analyses undertaken to evaluate the sensitivity of the findings, and investigate a number of interesting findings suitable for pursuit in further research. [file 12913_2019_4694_MOESM1_ESM.pdf]

Analysis notebook - Small is Beautiful? Explaining Resident Satisfaction in Swedish Nursing Home Care

Contents

Introduction . . . 1
Exploratory Factor analysis - Unit survey . . . 1
Exploratory Factor analysis - User survey . . . 5
Dropout analysis . . . 7
Descriptive statistics (Table 1) . . . 9
Regression diagnostics . . . 9
Regression models (Figure 1) . . . 12

Post-hoc analyses **17**
Non-linear effects . . . 17
Mediation analysis . . . 19
Associations with size . . . 20
Associations of user questions with composite measure . . . 21
Sub-group analysis - Questionnaire completion . . . 22
Alternate models . . . 23

Introduction

This document provides a reproducible accounting of the steps taken to generate the results presented in the manuscript "Small is Beautiful? Explaining Resident Satisfaction in Swedish Nursing Home Care", as well as a number of additional exploratory analyses performed to arrive at the final models presented in the paper, and post hoc analyses performed to further investigate effects identified in the main analysis.

The R code used to generate the tables, graphs, and model summaries presented here has been hidden for the sake of readability. To reproduce these results, see research data files available at https://data.mendeley.com/datasets/y69zhgxym3/draft?a=4ca98694-31d9-4685-af93-c3f6d7d2e51d (DOI here upon publication). In addition to the code used to generate this document, the repository also contains an interactive application which may be used to evaluate the sensitivity of our findings to alternate survey question weights. To run this application, we reccomend using RStudio (https://www.rstudio.com/) to open *app.R* in the *sensitivity_app* folder, and pressing the "Run App" button.

Exploratory Factor analysis - Unit survey

Let's examine our two nursing home-level datasets seperately first to identify internal patterns. A good first step is to investigate patterns with a correlation plot. Since many of the raw continuous variables are not normally distributed, we use Spearman rank correlations to characterize associations. We'll use complete case analysis here since we're only looking for general patterns. Since one question was missing for all short-term service facilities, we exclude these from the analysis.

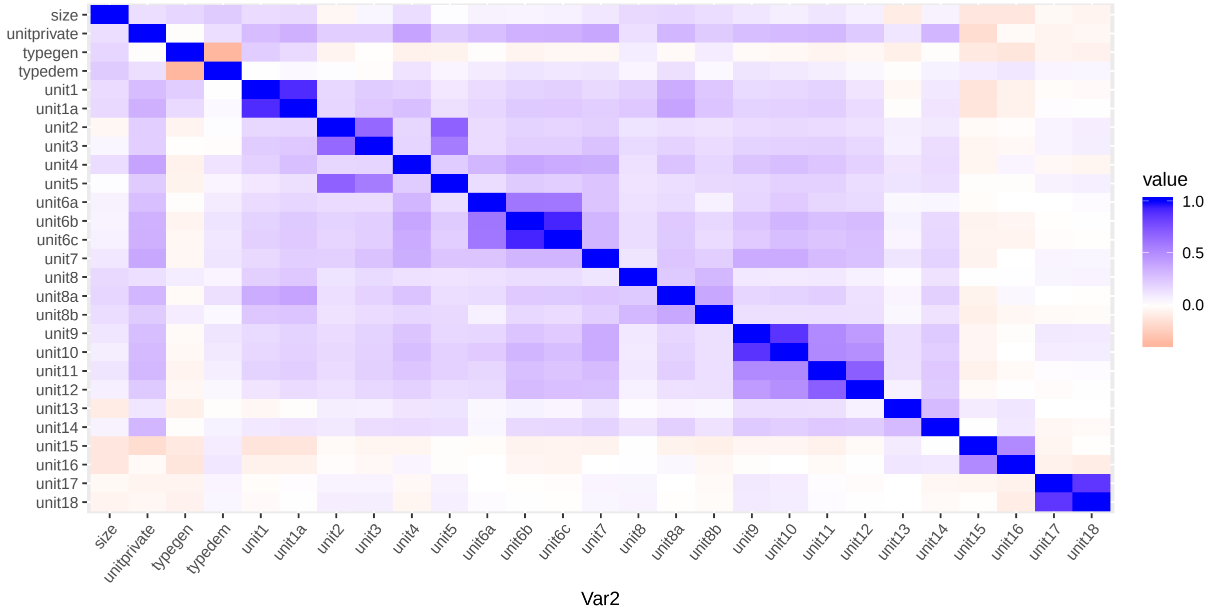

There is a lot to unpack here! Survey question descriptions may be found in a separate appendix, but generally, questions 1-7 assess for processes relating to individualized care, questions 8 and 8a-b assess for access to exercise and activities, questions 9-14 assess for processes related to patient safety, while questions 13-18 relate to staffing and education levels during the weekday and weekend.

To formalize our analysis, we performed a principal components analysis to help us decide how to proceed. Again, we use Spearman rank correlations to account for the heterogeneous distributions found in the data. We'll consider only the actual survey questions, as we have a strong theoretical basis for including the structural nursing home measures (Size, private ownership, and type of services provided) in our final analysis.

```
## [1] "Cronbachs alpha: 0.526720002433453"
```

Factor	Eigenvalue
1	5.1554025
2	2.0387944
3	1.9981374
4	1.7913008
5	1.7239732
6	1.4733257
7	1.1344544
8	1.0168535
9	0.8941221
10	0.7976697

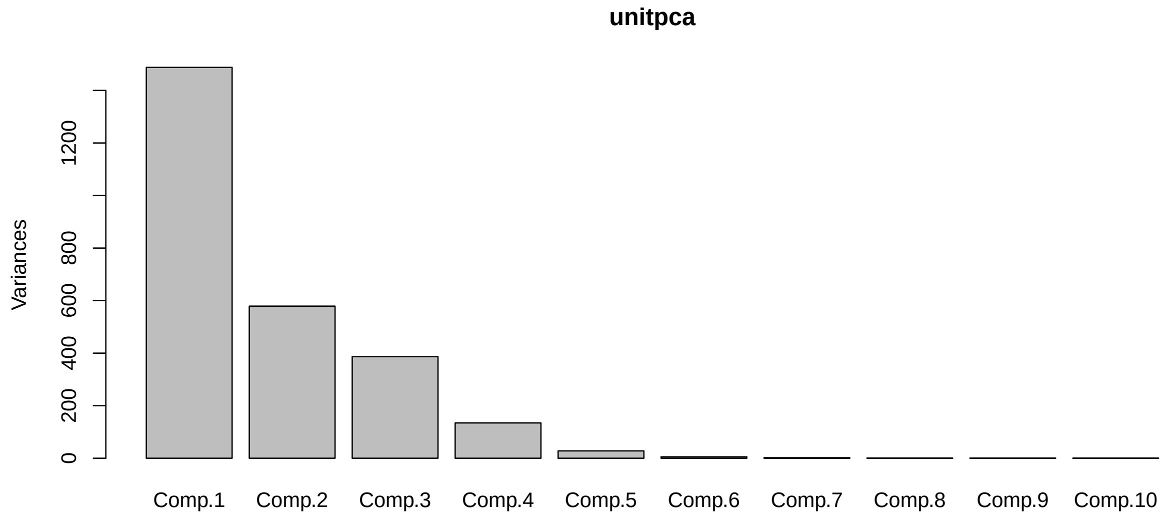

Based on the rule of thumb for an eigenvalue cutoff of 1, we find support for perhaps 7 or 8 factors in this data set, though the variance explained by these final factors becomes quite low. Let's see what a factor analysis can tell us. Note that we performed analyses using a range of rotation methods and factor counts, and readers are encouraged to experiment further with this data.

The NBHW groups these questions into 10 domains, which somewhat exceeds the number of components suggested by PCA, though we chose to retain these groups due to their conceptual value. Despite tinkering with optimization values, attempting to fit 10 factors resulted in a non-convergent model, so we present here an analysis based on 9 factors using varimax rotation.

```
##
## Call:
## factanal(x = ~., factors = 9, data = unitquestions, rotation = "varimax")
##
## Uniquenesses:
##  unit1 unit1a  unit2  unit3  unit4  unit5 unit6a unit6b unit6c  unit7
##  0.197  0.620  0.419  0.588  0.752  0.528  0.615  0.066  0.088  0.730
##  unit8 unit8a unit8b  unit9 unit10 unit11 unit12 unit13 unit14 unit15
##  0.829  0.608  0.574  0.072  0.173  0.442  0.005  0.402  0.672  0.005
## unit16 unit17 unit18
##  0.550  0.245  0.005
##
## Loadings:
##        Factor1 Factor2 Factor3 Factor4 Factor5 Factor6 Factor7 Factor8
## unit1                           0.105                   0.235   0.845
## unit1a  0.106                                           0.228   0.550
## unit2                           0.749
## unit3                           0.591                   0.133   0.155
## unit4   0.315   0.173           0.154                   0.247
## unit5   0.109                   0.651                   0.137
## unit6a  0.597
## unit6b  0.938   0.108                           0.104   0.137
## unit6c  0.931                                           0.122
## unit7   0.239   0.280           0.190           0.136   0.261
## unit8                           0.110                   0.368   0.112
```

```
## unit8a  0.123                                           0.550   0.237
## unit8b                                                  0.636
## unit9   0.119   0.926                           0.180
## unit10  0.182   0.839                           0.248
## unit11  0.139   0.374           0.105           0.589   0.129
## unit12  0.152   0.242                           0.949
## unit13
## unit14
## unit15                                  0.996
## unit16                 -0.116           0.655
## unit17                  0.859          -0.107
## unit18                  0.993
##        Factor9
## unit1
## unit1a
## unit2
## unit3
## unit4   0.121
## unit5
## unit6a
## unit6b
## unit6c
## unit7
## unit8
## unit8a
## unit8b
## unit9
## unit10
## unit11  0.121
## unit12
## unit13  0.764
## unit14  0.546
## unit15
## unit16
## unit17
## unit18
##
##                Factor1 Factor2 Factor3 Factor4 Factor5 Factor6 Factor7
## SS loadings      2.430   1.933   1.756   1.499   1.450   1.412   1.202
## Proportion Var   0.106   0.084   0.076   0.065   0.063   0.061   0.052
## Cumulative Var   0.106   0.190   0.266   0.331   0.394   0.456   0.508
##                Factor8 Factor9
## SS loadings      1.170   0.964
## Proportion Var   0.051   0.042
## Cumulative Var   0.559   0.601
##
## Test of the hypothesis that 9 factors are sufficient.
## The chi square statistic is 236.21 on 82 degrees of freedom.
## The p-value is 0.000000000000000073
```

See appendix 2 for a description of which variables were included in which conceptual categories. Generally, the questions loaded quite well only the categories proposed by the NBHW.

Exploratory Factor analysis - User survey

Here, we essentially redo the same steps with the user data. We'll have to exclude question 26 (who completed the questionnaire) due to the high rate of missingness (84%).

```
## [1] "Cronbachs alpha: 0.918047812530048"
```

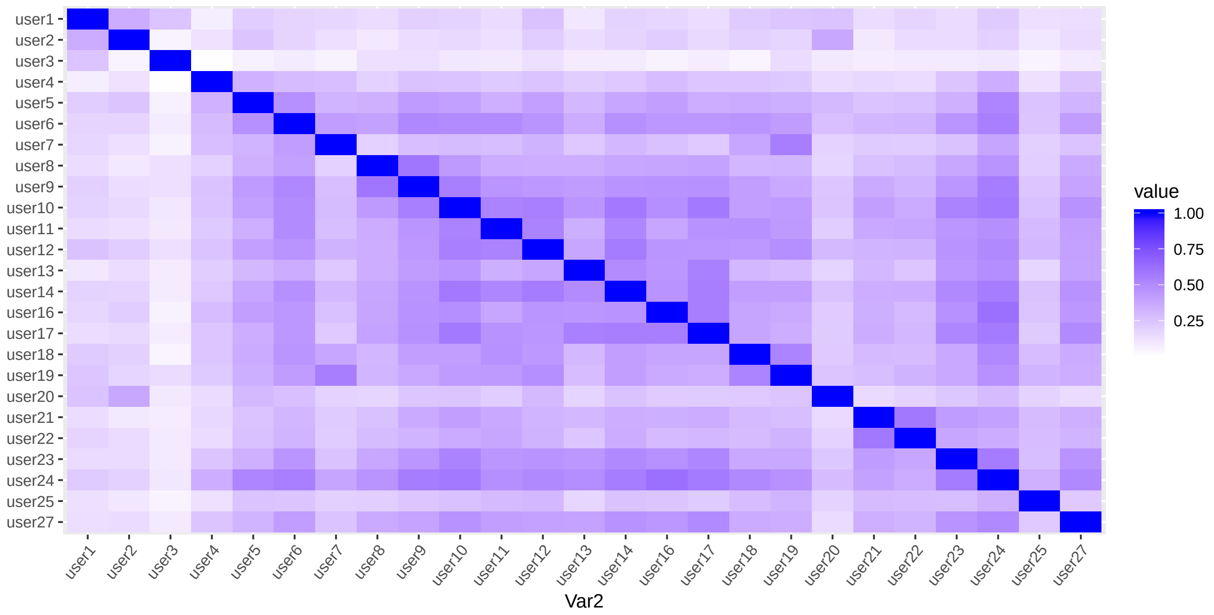

This doesn't bode well for extracting distinct factors. All the questions seem quite correlated, with only a few questions (1-3, 20, and 25) sticking out as less interrelated than the rest.

Factor	Eigenvalue
1	9.1431565
2	1.5495925
3	1.2163760
4	1.1931790
5	1.0536668
6	0.9159635
7	0.8437094
8	0.7778225
9	0.7330398
10	0.7059690

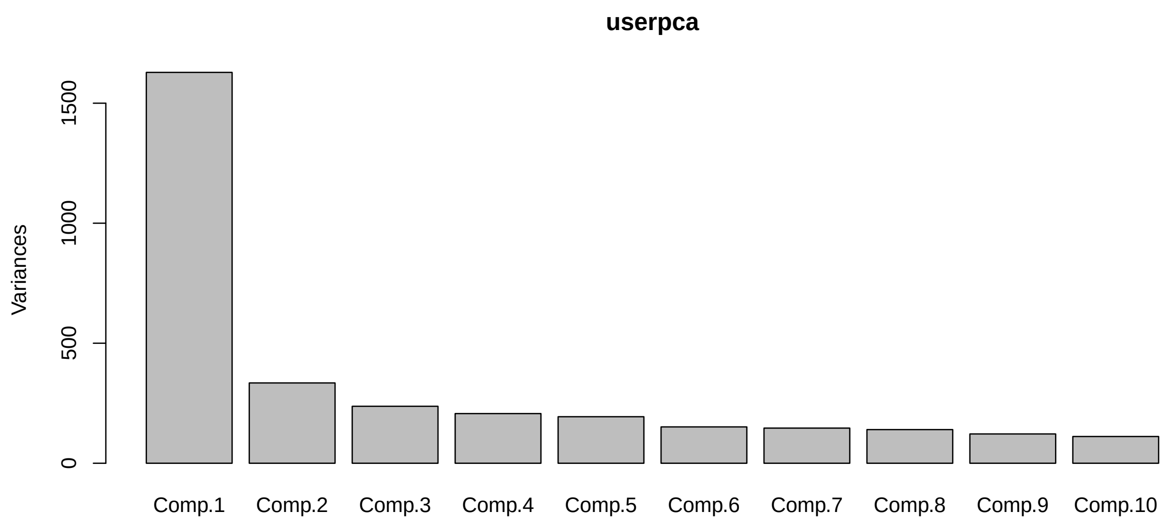

We see that the factor loadings drop quite dramatically down to just above an eigenvalue of 1. We chose to extract only 2 factors from this dataset, representing a measure of self-rated health (Questions 1-3 and 20), and an aggregate measure of satisfaction (the remainder sans question 26 which had 84% missing values). We opted to make this distinction based on theory in light of the highly colinear nature of this dataset, but for completeness, here are the factor loadings assuming 2 factors.

```
##
## Loadings:
##        Factor1 Factor2
## user1  0.110   0.362
## user2  0.116   0.292
## user3          0.157
## user4  0.311   0.281
## user5  0.426   0.428
## user6  0.497   0.491
## user7  0.162   0.617
## user8  0.533   0.231
## user9  0.616   0.331
## user10 0.688   0.350
## user11 0.506   0.424
## user12 0.509   0.487
## user13 0.666   0.134
## user14 0.656   0.339
## user16 0.652   0.281
## user17 0.762   0.186
## user18 0.366   0.571
## user19 0.261   0.713
## user20 0.199   0.364
## user21 0.449   0.259
## user22 0.376   0.313
## user23 0.669   0.257
## user24 0.696   0.422
## user25 0.268   0.338
## user27 0.613   0.234
```

```
##
##                Factor1 Factor2
## SS loadings      6.076   3.618
## Proportion Var   0.243   0.145
## Cumulative Var   0.243   0.388
```

It may be noted that these loadings are quite sensitive to changes in rotation and number of factors. It may also be noted that the overall correlations found in this survey were quite strong, as suggested by a

Dropout analysis

Of the 2088 nursing homes in the unit survey, and the 1921 homes in the user survey, we were able to successfully match 1798 of these (86% and 93% of the homes reported in each respective dataset) to create a combined dataset. One potential source of bias is differences in variables associated with not being matched. Let's take a look at how our variables differ between matched and non-matched NHs.

First for the unit survey:

	Matched					Not Matched					
	Mean	SD	Median	IQR	Missing	Mean	SD	Median	IQR	Missing	U-test P-value
Size of nursing home	43.57	22.70	39.00	25.00	6	18.21	11.72	16.00	12.00	1	0.000
Private ownership per Unit Survey	0.19	0.39	0.00	0.00	0	0.14	0.35	0.00	0.00	0	0.049
Has general care facilities	0.79	0.41	1.00	0.00	0	0.58	0.49	1.00	1.00	0	0.000
Has dementia care facilities	0.59	0.49	1.00	1.00	0	0.53	0.50	1.00	1.00	0	0.064
Has assisted living facilities	0.05	0.23	0.00	0.00	0	0.03	0.18	0.00	0.00	0	0.163
Participation in resident councils	0.04	0.87	0.46	1.41	0	-0.23	0.91	-0.95	1.41	0	0.000
Individualized action plans	-0.01	0.92	0.43	0.92	0	-0.11	1.05	0.50	1.34	0	0.917
Patient safety routines	0.02	0.80	-0.26	1.59	0	-0.12	0.73	-0.26	1.07	0	0.014
Availability of exercise and activity	0.05	0.81	0.12	1.48	0	-0.33	0.90	-0.18	1.10	0	0.000
Care coordination routines	0.02	0.97	-0.03	2.00	0	-0.13	0.96	-1.03	2.00	0	0.012
Medication review routines	0.02	0.92	0.11	2.01	0	-0.15	0.89	-0.89	2.01	0	0.003
Staff per resident	0.29	0.06	0.28	0.06	41	0.31	0.08	0.30	0.07	5	0.000
Staff with adequate education	83.71	14.12	86.86	18.54	40	82.29	17.19	85.57	25.14	5	0.940
match	1.00	0.00	1.00	0.00	0	0.00	0.00	0.00	0.00	0	0.000

And then for the User survey

	Matched					Not Matched					
	Mean	SD	Median	IQR	Missing	Mean	SD	Median	IQR	Missing	U-test P-value
Response rate to User Survey	0.57	0.12	0.50	0.20	0	0.55	0.12	0.50	0.20	0	0.114
Aggregate resident satisfaction	0.01	1.00	0.05	1.34	4	-0.11	1.03	-0.03	1.45	0	0.270
Aggregate Self-Rated Health	0.01	1.00	-0.04	1.31	12	-0.18	1.03	-0.24	1.34	3	0.036
match	1.00	0.00	1.00	0.00	0	0.00	0.00	0.00	0.00	0	0.000

It appears that non-matched nursing homes are quite a bit smaller than matched homes, score quite a bit lower on process-related measures, and have fewer opportunities for physical activity. In terms of the satisfaction survey, We find that non-matched homes have perhaps slightly lower self-rated health and satisfaction than their matched counterparts. As demonstrated using Mann-Whitney U tests, several differences in the Unit survey items noted here are significant, while only the self rated health variable in the user survey may be shown to differ significantly between matched and non-matched nursing homes.

Another source of missingness in this data is non-response to the user survey. To investigate potential biases caused by non-response, a model was specified to identify variables associated with the survey response rate. Note that while these data were presented in binned form (i.e. response rates between 0% - 40% were grouped together), we used a linear model as these respresented an underlying continuous and apparently normal distribution.

	Estimate	2.5 %	97.5 %
Intercept	-0.021	-0.085	0.042
Aggregate resident satisfaction	0.136	0.084	0.186
Participation in resident councils	0.034	-0.021	0.086
Individualized action plans	0.041	-0.013	0.100
Meal-related routines and plans	-0.012	-0.068	0.048
Patient safety routines	0.005	-0.052	0.062
Care coordination routines	-0.015	-0.081	0.046
Medication review routines	-0.016	-0.077	0.042
Availability of exercise and activity	0.039	-0.019	0.097
Size of nursing home	-0.136	-0.189	-0.082
Private ownership	0.071	0.015	0.126
Nurses per resident	-0.005	-0.055	0.048
Staff per resident	-0.028	-0.076	0.026
Staff with adequate education	-0.026	-0.075	0.027
Has general care facilities	0.136	0.083	0.190
Has dementia care facilities	-0.104	-0.159	-0.056
Has assisted living facilities	0.100	0.028	0.178
Aggregate Self-Rated Health	0.015	-0.035	0.068
Population 65+ in Nursing Home (%)	-0.033	-0.104	0.038
Population 65+ (%)	-0.118	-0.198	-0.049
Population per square kilometer	-0.083	-0.174	0.029
Average annual cost per resident (SEK)	-0.045	-0.107	0.024
Average age of residents in nursing homes	0.049	-0.011	0.109
Political control (left = -1, mixed = 0, right = 1)	-0.001	-0.075	0.077
Average annual per capita taxable income (SEK)	0.036	-0.039	0.106

We find that among the variables we are interested in estimating, response rates are associated with resident satisfaction, negatively associated with nursing home size, and positively associated with private ownership of the nursing homes. The implications of these findings are discussed in the manuscript.

Descriptive statistics (Table 1)

Now that we have a grip on these datasets, lets take a look at our combined dataset. Let's begin with some descriptive data for the aggregated measures which we developed based on our exploratory analysis. This is Table 1 in the manuscript

	Mean	SD	Median	IQR	Missing
Aggregate resident satisfaction	0.01	1.00	0.05	1.34	4
Participation in resident councils	0.00	1.00	0.48	1.63	0
Individualized action plans	0.00	1.00	0.48	1.00	0
Meal-related routines and plans	0.00	1.00	-0.17	1.60	53
Patient safety routines	0.00	1.00	-0.35	1.98	0
Care coordination routines	0.00	1.00	-0.05	2.07	0
Medication review routines	0.00	1.00	0.09	2.18	0
Availability of exercise and activity	0.00	1.00	0.08	1.82	0
Private ownership	0.19	0.39	0.00	0.00	1
Size of nursing home	43.57	22.70	39.00	25.00	6
Nurses per resident	0.03	0.01	0.03	0.02	62
Staff per resident	0.29	0.06	0.28	0.06	41
Staff with adequate education	83.71	14.12	86.86	18.54	40
Has general care facilities	0.79	0.41	1.00	0.00	0
Has dementia care facilities	0.59	0.49	1.00	1.00	0
Has assisted living facilities	0.05	0.23	0.00	0.00	0
Aggregate Self-Rated Health	0.01	1.00	-0.04	1.31	12
Population 65+ in Nursing Home (%)	4.21	0.88	4.21	0.99	19
Population 65+ (%)	21.22	4.19	21.20	6.33	0
Population per square kilometer	472.49	1164.71	60.62	116.03	0
Average annual cost per resident (SEK)	838285.24	161812.23	822686.24	117267.27	19
Average age of residents in nursing homes	83.49	1.82	83.60	2.30	0
Political control (left = -1, mixed = 0, right = 1)	-0.12	0.80	0.00	2.00	0
Average annual per capita taxable income (SEK)	188232.40	24921.26	183269.64	23691.47	0

Regression diagnostics

Let's first check some model assumptions for a simple linear regression consisting of all our predictor variables. While we'll be using a few different models in our analysis, this should give us a good picture of what to look out for.

QQ-plot of residuals

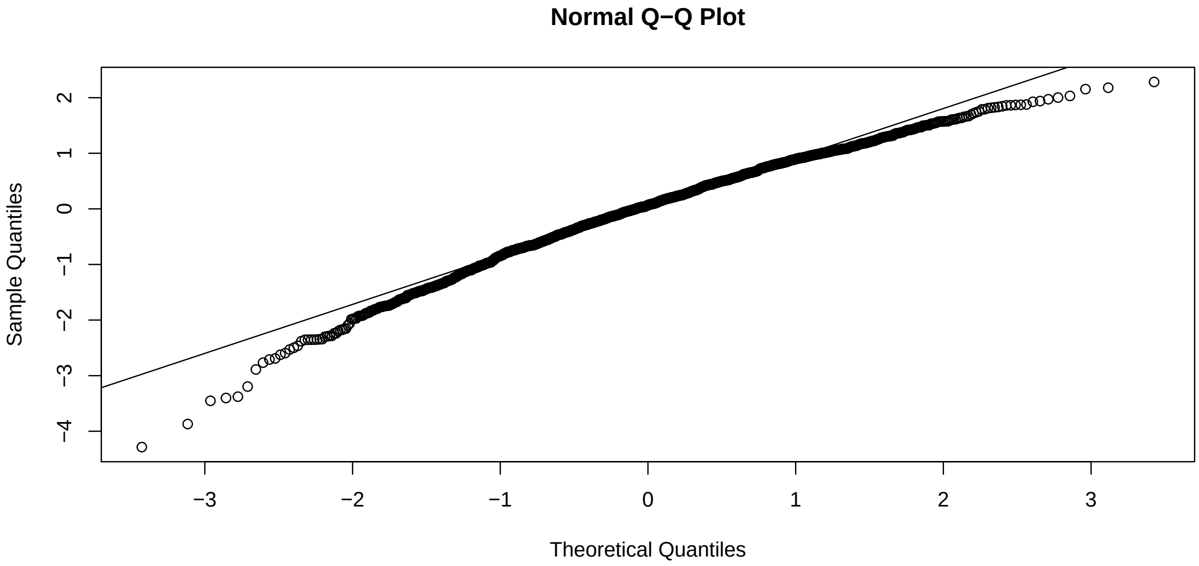

Plot of residuals v. fitted values

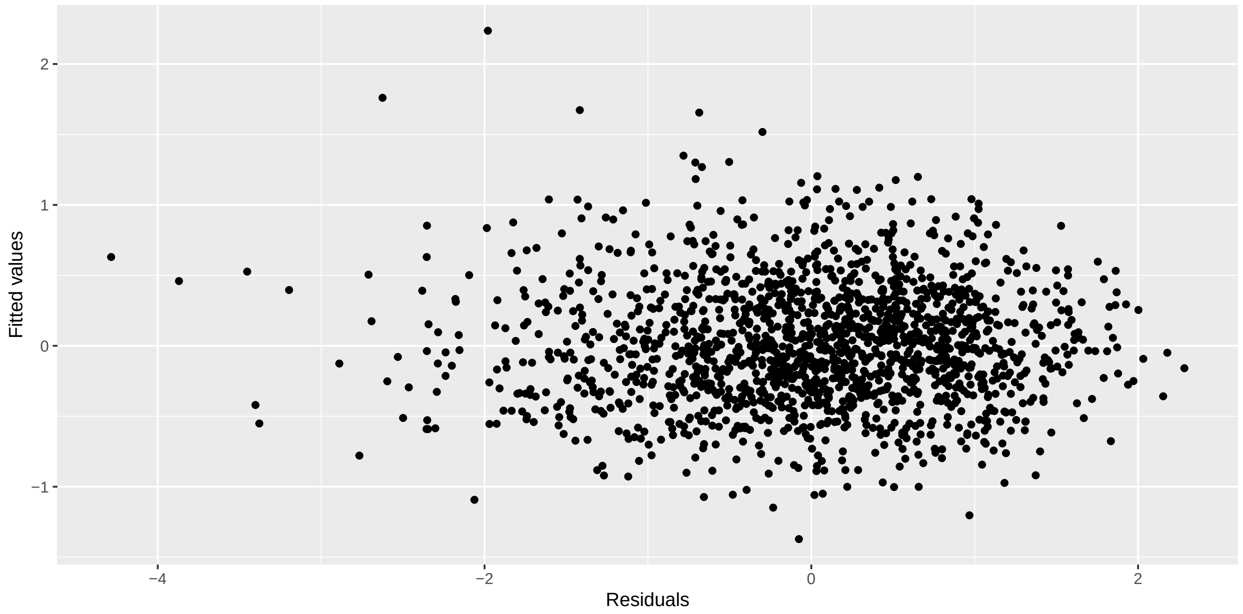

Breusch-Pagan test of heteroskedasticity:

```
##
##  studentized Breusch-Pagan test
##
## data:  lmfit
## BP = 33.613, df = 16, p-value = 0.006123
```

Variable Inflation Factors:

```
## residentcouncil      actionplan           meals  safetyroutines
##        1.269672        1.318307        1.604375        1.507199
```

```
##       carecoord       medreview        activity         private
##        1.524005        1.464741        1.290086        1.445695
##            size             rns           staff             edu
##        1.122483        1.061378        1.049314        1.041220
##         typegen         typedem        typeserv          srhtot
##        1.208869        1.233964        1.035926        1.019041
```

Bootstrap validation results:

```
##           index.orig training   test optimism index.corrected   n
## R-square      0.1898   0.1970 0.1810   0.0160          0.1737 200
## MSE           0.8069   0.8021 0.8157  -0.0135          0.8204 200
## g             0.4860   0.4958 0.4766   0.0192          0.4668 200
## Intercept     0.0000   0.0000 0.0014  -0.0014          0.0014 200
## Slope         1.0000   1.0000 0.9637   0.0363          0.9637 200
```

While we don't seem to have problems with overfitting, we do have some outliers which could affect inferences in a linear model assuming normally distributed residuals. While mutlicolinearity is below typically accepted thresholds, for some of the process measures, the variable inflation factor is high enough that it could cast some doubt on the interpretation of our results. To avoid this, we'll estimate each predictor variable independently.

Our model also has some trouble with heteroskedasticity per the Breusch-Pagan test, and a visual inspection of residuals reveals some potential influential outliers. It seems likely that this is due to the skew in outcome data, and as such is likely to be an issue in more restricted models as well. To deal with this, we chose to use the Huber-White sandwich estimator to provide consistent coefficient estimates..

Next, let's take a look at our heirachial models. We developed our models based on theory, and we're primarily interested in estimating fixed effects, but getting a sense of inter- and intra- municipality variation is quite interesting, and it's always a good idea to verify that the variables we're adding actually contribute to a good model fit.

Check intra-class correlation:

```
## # Intraclass Correlation Coefficient
##
##      Adjusted ICC: 0.100
##   Conditional ICC: 0.100
```

ANOVA test to check for model superiority:

```
## Data: compaggdata
## Models:
## mlnull: sattot ~ 1 + (1 | munin)
## mlnh: sattot ~ residentcouncil + actionplan + meals + safetyroutines +
## mlnh:     carecoord + medreview + activity + private + size + rns +
## mlnh:     staff + edu + typegen + typedem + typeserv + srhtot + (1 |
## mlnh:     munin)
##        Df    AIC    BIC  logLik deviance Chisq Chi Df
## mlnull  3 4481.4 4497.5 -2237.7   4475.4
## mlnh   19 4181.6 4283.8 -2071.8   4143.6 331.8     16
##                   Pr(>Chisq)
## mlnull
## mlnh   < 0.00000000000000022 ***
## ---
## Signif. codes:  0 '***' 0.001 '**' 0.01 '*' 0.05 '.' 0.1 ' ' 1
```

```
## Data: compaggdata
## Models:
## mlnh: sattot ~ residentcouncil + actionplan + meals + safetyroutines +
```

```
## mlnh:     carecoord + medreview + activity + private + size + rns +
## mlnh:     staff + edu + typegen + typedem + typeserv + srhtot + (1 |
## mlnh:     munin)
## mlnhmuni: sattot ~ residentcouncil + actionplan + meals + safetyroutines +
## mlnhmuni:     carecoord + medreview + activity + private + size + rns +
## mlnhmuni:     staff + edu + typegen + typedem + typeserv + srhtot + pop65innh +
## mlnhmuni:     pop65 + popkm + costperpt + nhage + polcontrol + taxpower +
## mlnhmuni:     (1 | munin)
##           Df    AIC    BIC  logLik deviance  Chisq Chi Df  Pr(>Chisq)
## mlnh      19 4181.6 4283.8 -2071.8   4143.6
## mlnhmuni 26 4156.1 4295.9 -2052.1   4104.1 39.487      7 0.000001577 ***
## ---
## Signif. codes:  0 '***' 0.001 '**' 0.01 '*' 0.05 '.' 0.1 ' ' 1
```

QQ plot of random effect residuals:

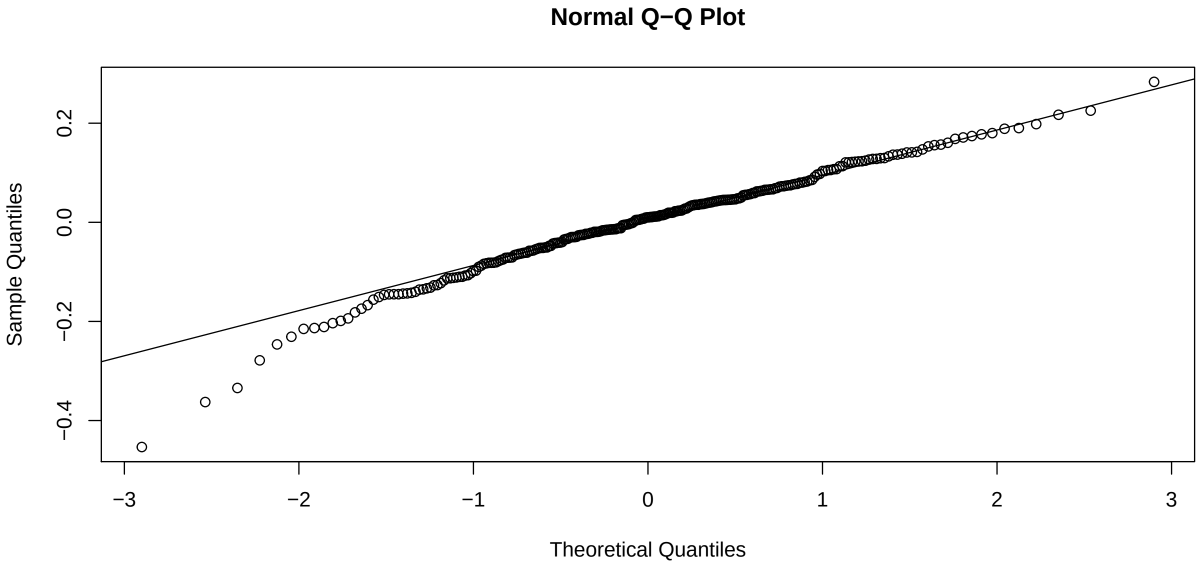

We can interpret the ICC as indicating that 9.9% of the variation in satisfaction occurs at the municipality level. Based on ANOVA results, we find that including both the nursing home level and municipality level fixed effects contribute to a good model fit. Note that while we tried fitting some models with random slopes as well, many municipalities lack a sufficient sample size for this approach to (in our attempts) produce reliable results. We see that there is some deviation from normality in the sparse lower quantiles, but this seems close enough to generate valid inferences, especially given the use of bootstrapping to generate confidence intervals.

Regression models (Figure 1)

Let's go ahead and print each of the coefficient estimates reported in figure 1, and construct the figure included in the article:

fw	var	group	type	Bivariate	Health controlled	Health and Structure controlled

1a - Classical OLS Regression	Participation in resident councils	Individualized care	Processual measures	0.051 (0.004 - 0.097)	0.031 (-0.013 - 0.074)	0.048 (0.003 - 0.094)
1a - Classical OLS Regression	Individualized action plans	Individualized care	Processual measures	0.067 (0.016 - 0.117)	0.077 (0.032 - 0.123)	0.074 (0.027 - 0.122)
1a - Classical OLS Regression	Meal-related routines and plans	Individualized care	Processual measures	0.046 (-0.005 - 0.096)	0.048 (0.001 - 0.095)	0.043 (-0.008 - 0.094)
1a - Classical OLS Regression	Patient safety routines	Safe care	Processual measures	-0.011 (-0.058 - 0.036)	-0.006 (-0.049 - 0.037)	-0.022 (-0.072 - 0.028)
1a - Classical OLS Regression	Care coordination routines	Safe care	Processual measures	0.002 (-0.045 - 0.048)	-0.005 (-0.048 - 0.039)	-0.02 (-0.066 - 0.026)
1a - Classical OLS Regression	Medication review routines	Safe care	Processual measures	0.005 (-0.041 - 0.052)	0.004 (-0.038 - 0.047)	-0.002 (-0.047 - 0.043)
1a - Classical OLS Regression	Availability of exercise and activity	Activity	Processual measures	0.083 (0.035 - 0.131)	0.078 (0.033 - 0.122)	0.108 (0.06 - 0.156)
1a - Classical OLS Regression	Private ownership	Ownership	Structural measures	0.024 (-0.024 - 0.072)	0.015 (-0.03 - 0.06)	0.028 (-0.018 - 0.074)
1a - Classical OLS Regression	Size of nursing home	Size	Structural measures	-0.197 (-0.241 - -0.154)	-0.176 (-0.217 - -0.135)	-0.181 (-0.226 - -0.136)
1a - Classical OLS Regression	Nurses per resident	Staffing	Structural measures	0.043 (-0.006 - 0.091)	0.039 (-0.007 - 0.085)	0.011 (-0.036 - 0.057)
1a - Classical OLS Regression	Staff per resident	Staffing	Structural measures	0.066 (0.02 - 0.111)	0.087 (0.044 - 0.13)	0.069 (0.023 - 0.115)
1a - Classical OLS Regression	Staff with adequate education	Staffing	Structural measures	0.054 (0.005 - 0.103)	0.067 (0.022 - 0.112)	0.059 (0.013 - 0.106)
1a - Classical OLS Regression	Has general care facilities	NA	NA	-0.036 (-0.084 - 0.011)	NA	NA

1a - Classical OLS Regression	Has dementia care facilities	NA	NA	0.032 (-0.014 - 0.079)	NA	NA
1a - Classical OLS Regression	Has assisted living facilities	NA	NA	0.027 (-0.009 - 0.064)	NA	NA
1a - Classical OLS Regression	Aggregate Self-Rated Health	NA	NA	0.363 (0.318 - 0.409)	NA	NA
1b - Mixed-Effects regression with Municipal-level control variables	Participation in resident councils	Individualized care	Processual measures	0.069 (0.017 - 0.117)	0.051 (0.005 - 0.094)	0.062 (0.01 - 0.109)
1b - Mixed-Effects regression with Municipal-level control variables	Individualized action plans	Individualized care	Processual measures	0.071 (0.021 - 0.12)	0.085 (0.039 - 0.131)	0.071 (0.026 - 0.116)
1b - Mixed-Effects regression with Municipal-level control variables	Meal-related routines and plans	Individualized care	Processual measures	0.072 (0.016 - 0.119)	0.078 (0.029 - 0.121)	0.054 (0 - 0.104)
1b - Mixed-Effects regression with Municipal-level control variables	Patient safety routines	Safe care	Processual measures	0.014 (-0.033 - 0.065)	0.017 (-0.028 - 0.064)	-0.012 (-0.061 - 0.037)
1b - Mixed-Effects regression with Municipal-level control variables	Care coordination routines	Safe care	Processual measures	0.029 (-0.023 - 0.079)	0.021 (-0.026 - 0.067)	-0.002 (-0.05 - 0.052)
1b - Mixed-Effects regression with Municipal-level control variables	Medication review routines	Safe care	Processual measures	0.026 (-0.021 - 0.075)	0.024 (-0.024 - 0.073)	0.002 (-0.051 - 0.049)

1b - Mixed-Effects regression with Municipal-level control variables	Availability of exercise and activity	Activity	Processual measures	0.114 (0.067 - 0.17)	0.109 (0.064 - 0.153)	0.122 (0.072 - 0.17)
1b - Mixed-Effects regression with Municipal-level control variables	Private ownership	Ownership	Structural measures	0.078 (0.026 - 0.128)	0.064 (0.008 - 0.118)	0.064 (0.016 - 0.113)
1b - Mixed-Effects regression with Municipal-level control variables	Size of nursing home	Size	Structural measures	-0.184 (-0.232 - -0.139)	-0.159 (-0.204 - -0.117)	-0.153 (-0.195 - -0.102)
1b - Mixed-Effects regression with Municipal-level control variables	Nurses per resident	Staffing	Structural measures	0.071 (0.022 - 0.117)	0.063 (0.018 - 0.113)	0.031 (-0.017 - 0.077)
1b - Mixed-Effects regression with Municipal-level control variables	Staff per resident	Staffing	Structural measures	0.056 (0.009 - 0.106)	0.072 (0.025 - 0.115)	0.06 (0.017 - 0.105)
1b - Mixed-Effects regression with Municipal-level control variables	Staff with adequate education	Staffing	Structural measures	0.052 (0.003 - 0.104)	0.065 (0.021 - 0.113)	0.061 (0.015 - 0.106)
1b - Mixed-Effects regression with Municipal-level control variables	Has general care facilities	NA	NA	-0.054 (-0.101 - -0.011)	NA	NA
1b - Mixed-Effects regression with Municipal-level control variables	Has dementia care facilities	NA	NA	0.047 (0 - 0.094)	NA	NA

1b - Mixed-Effects regression with Municipal-level control variables	Has assisted living facilities	NA	NA	0.019 (-0.032 - 0.067)	NA	NA
1b - Mixed-Effects regression with Municipal-level control variables	Aggregate Self-Rated Health	NA	NA	0.354 (0.312 - 0.397)	NA	NA

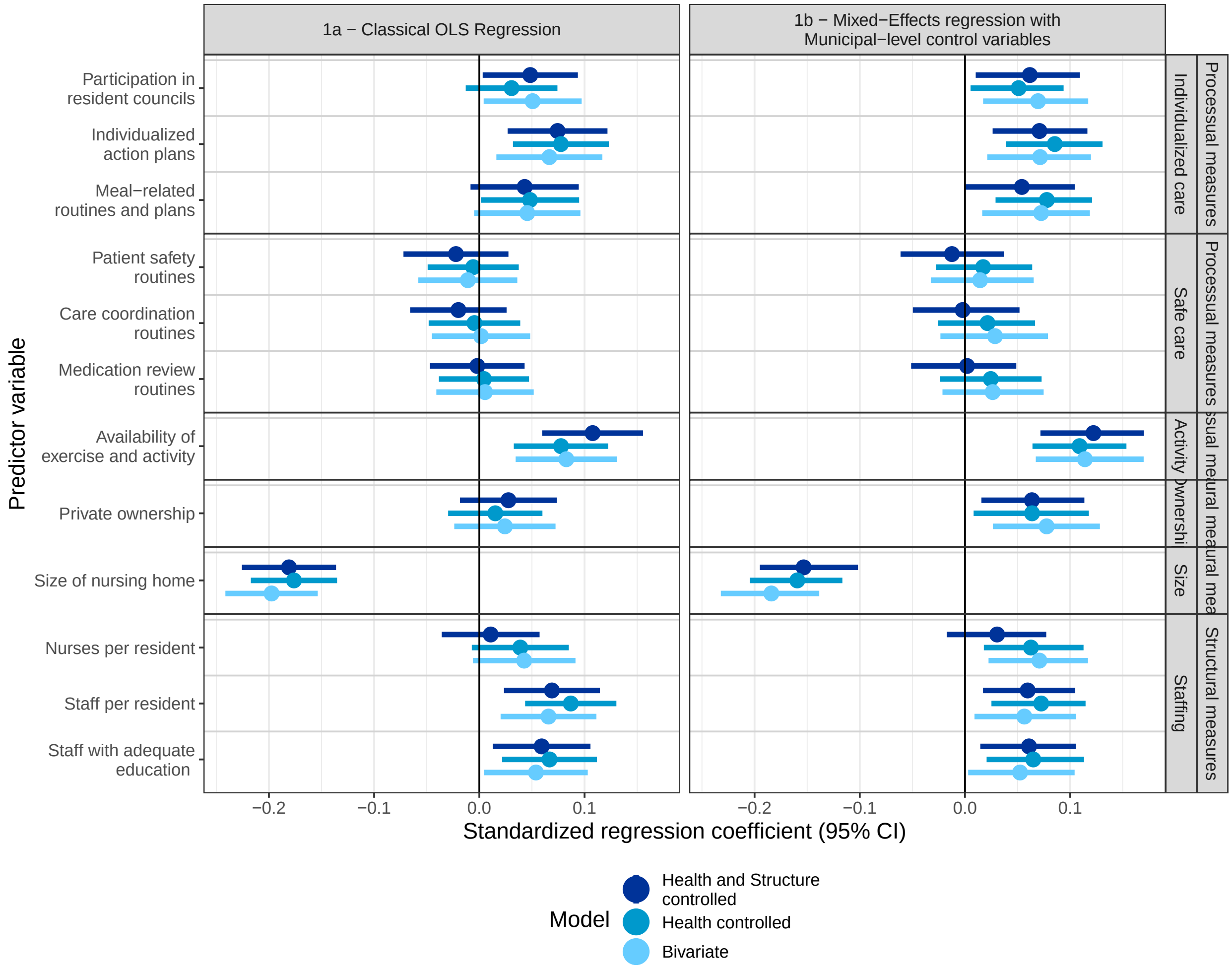

These results are discussed in detail in the manuscript. Lets also go ahead and print out the full list of model coefficients for the full multi-level model including municipal level controls. Note that these differ slightly from the data reported in the manuscript - We chose to control for confounding effects in a somewhat more restricted manner than simply including every predictor in a multivariable model.

		2.5 %	97.5 %
(Intercept)	0.014	-0.041	0.074
residentcouncil	0.035	-0.016	0.083
actionplan	0.044	-0.011	0.098
meals	0.027	-0.034	0.080
safetyroutines	-0.042	-0.093	0.015
carecoord	-0.008	-0.067	0.051
medreview	-0.006	-0.070	0.045
activity	0.110	0.061	0.163
private	0.036	-0.015	0.092
size	-0.168	-0.215	-0.121
rns	0.029	-0.018	0.080
staff	0.055	0.010	0.105
edu	0.056	0.008	0.101
typegen	-0.032	-0.085	0.017
typedem	0.061	0.009	0.114
typeserv	0.019	-0.047	0.086
srhtot	0.345	0.300	0.389
pop65innh	-0.033	-0.096	0.031
pop65	0.049	-0.024	0.118
popkm	-0.011	-0.108	0.096
costperpt	-0.011	-0.076	0.049
nhage	0.096	0.040	0.155
polcontrol	0.068	-0.007	0.145
taxpower	-0.083	-0.156	-0.008

Post-hoc analyses

Non-linear effects

While we chose to assume linearity in our reported models to provide a more intuitive interpretation of our results, we did assess for non-linear effects using restricted cubic splines with interesting results. Since we're only interested in the form of the spline, and not the absolute effect of the variable here, we can load all of our variables into a single model for ease of analysis.

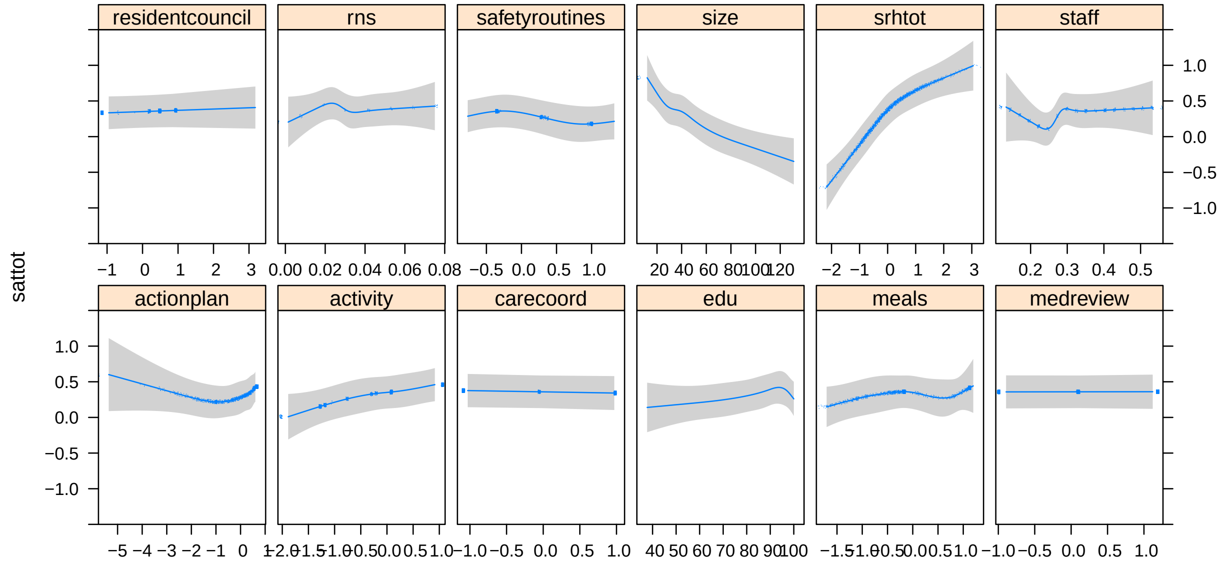

We see that some variables display interesting patterns using this technique. Some are not readily interpretable, but two in particular stand out as candidates for further investigation, namely the variables for staff education and non-nurse staffing levels:

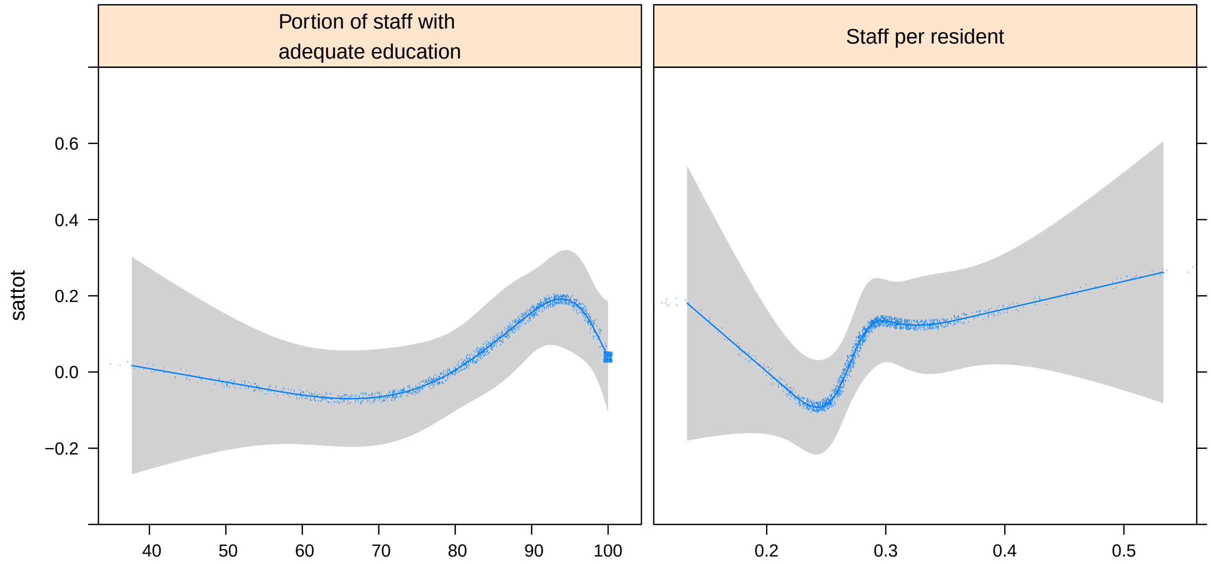

Here we see that for staff education, satisfaction drops from a peak around 94% with an "adequate" level of training to a lower level of satisfaction for sites reporting 100% "adequately educated" staff. We also see some suggestion of a threshold effect for staffing levels, with diminishing returns after increasing staffing ratios beyond 0.3 staff per resident. These effects are not quite significant, and performing detailed post hoc analysis is likely to lead to high "researcher degrees of freedom" - as such we leave these findings to be pursued in further research.

Mediation analysis

To investigate potential mediation effects at the nursing home level, we performed an analysis of average causal mediation effects (ACME) between each of the process and structure measures.

measure	mediator	est	low	high	p
size	residentcouncil	-0.036	-0.071	-0.014	0.02
size	actionplan	-0.017	-0.039	-0.004	0.02
size	meals	-0.026	-0.058	-0.003	0.00
size	safetyroutines	-0.001	-0.013	0.013	0.90
size	carecoord	-0.006	-0.028	0.014	0.40
size	medreview	-0.002	-0.019	0.015	0.76
size	activity	-0.095	-0.170	-0.051	0.00
staff	residentcouncil	-0.092	-0.505	0.015	0.08
staff	actionplan	0.001	-0.197	0.123	0.98
staff	meals	0.002	-0.075	0.111	0.80
staff	safetyroutines	0.003	-0.087	0.058	0.96
staff	carecoord	-0.003	-0.081	0.046	0.84
staff	medreview	-0.002	-0.118	0.078	0.86
staff	activity	-0.110	-1.429	0.102	0.14
rns	residentcouncil	0.055	-0.453	0.342	0.28
rns	actionplan	0.112	-0.750	1.163	0.22
rns	meals	0.147	-1.535	1.427	0.24
rns	safetyroutines	-0.034	-0.545	0.208	0.58
rns	carecoord	-0.017	-0.465	0.756	0.86
rns	medreview	-0.020	-0.363	0.782	0.82
rns	activity	0.232	-2.582	0.928	0.16
edu	residentcouncil	-0.008	-0.111	0.113	0.74
edu	actionplan	0.089	0.016	0.509	0.04
edu	meals	0.011	-0.022	0.078	0.58
edu	safetyroutines	0.001	-0.073	0.045	0.94
edu	carecoord	-0.002	-0.083	0.158	0.96
edu	medreview	0.000	-0.024	0.078	0.88
edu	activity	0.005	-0.142	0.115	0.96
private	residentcouncil	0.580	-11.347	4.554	0.42
private	actionplan	0.523	-6.216	4.229	0.28
private	meals	0.635	-5.092	5.022	0.28
private	safetyroutines	-0.355	-4.837	3.242	0.46
private	carecoord	-0.073	-2.342	2.024	0.98
private	medreview	-0.100	-5.205	0.796	0.72
private	activity	1.238	-5.673	11.879	0.38

We see that by and large, the mediating effects in this data are quite weak, as may be expected in a dataset such as this with quite weak overall effects. Let’s filter this using a p-value of 0.05 as a cutoff and plot the results:

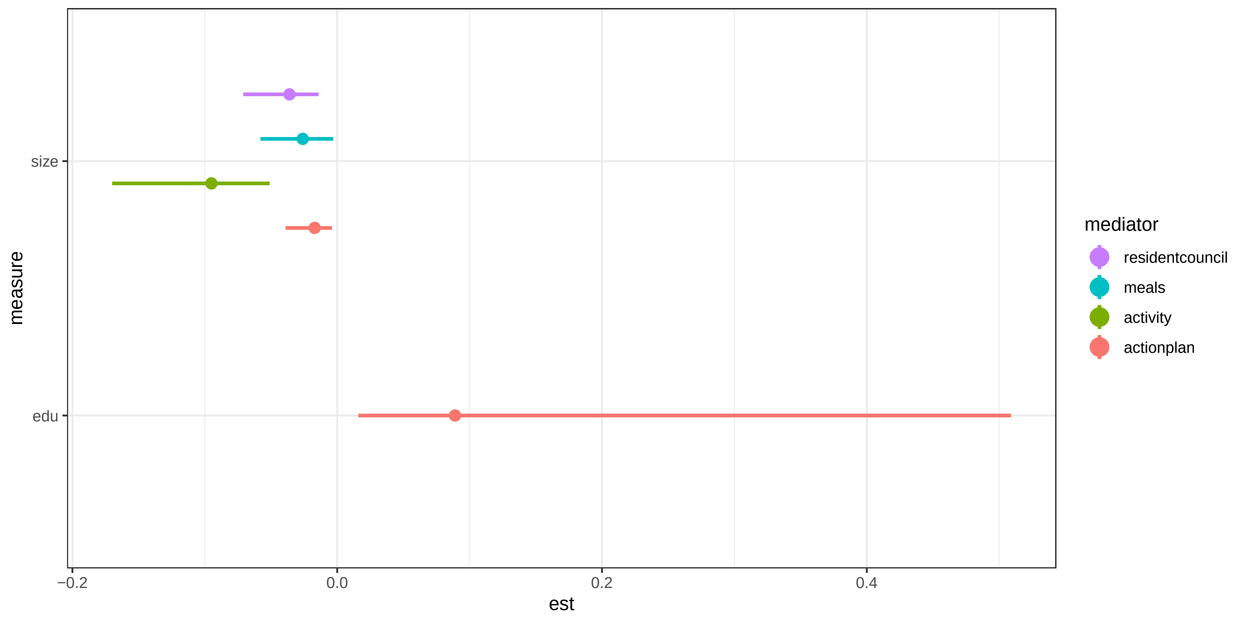

We found significant mediating effects with regards to nursing home size, with the most pronounced effects found with regards to exercise and activity. This suggests that the negative effect on satisfaction of larger nursing homes is to some extent mediated by the provision of more activities and individualized care processes - in other words, larger nursing homes provide more activities, explaining the increase in importance of the activity variable upon controlling for structural variables.

Associations with size

As it was thought that there may be important differences with regards to conditions across various sizes of nuring homes, we investigated the average values of the analytical and control variables across various sizes of nuring homes, here binned by quintiles (i.e. 5 groups):

	1	2	3	4	5
N	345	347	373	360	367
Size range	8 - 25	26 - 33	34 - 42	43 - 56	57 - 176
Participation in resident councils	-0.102	-0.023	0.043	0.067	0.180
Individualized action plans	-0.085	0.002	-0.013	0.034	0.059
Meal-related routines and plans	-0.093	-0.058	0.004	0.145	0.130
Patient safety routines	-0.035	-0.054	0.024	0.045	0.113
Care coordination routines	0.013	-0.105	-0.006	0.050	0.133
Medication review routines	-0.012	-0.093	0.067	0.032	0.124
Availability of exercise and activity	-0.064	-0.103	0.014	0.178	0.220
Private ownership	0.142	0.147	0.166	0.242	0.240
Size of nursing home	20.322	29.548	38.091	49.089	78.850
Nurses per resident	0.035	0.033	0.034	0.032	0.031
Staff per resident	0.294	0.288	0.296	0.284	0.277
Staff with adequate education	82.838	84.693	83.689	84.038	83.323
Has general care facilities	0.739	0.755	0.788	0.833	0.815
Has dementia care facilities	0.449	0.519	0.579	0.694	0.711
Has assisted living facilities	0.032	0.046	0.062	0.044	0.084
Aggregate Self-Rated Health	0.118	0.057	-0.019	-0.035	-0.052
Population 65+ in Nursing Home (%)	4.128	4.216	4.216	4.242	4.264
Population 65+ (%)	22.378	22.151	21.466	20.646	19.630
Population per square kilometer	291.133	288.278	347.298	642.971	757.960
Average annual cost per resident (SEK)	856804.5	835606.0	848870.4	832273.1	815463.9
Average age of residents in nursing homes	83.776	83.414	83.504	83.463	83.329
Political control (left = -1, mixed = 0, right = 1)	-0.157	-0.101	-0.075	-0.142	-0.131
Average annual per capita taxable income (SEK)	182433.4	183177.8	187857.9	190768.6	196218.7

As seen also in the mediation analysis, we find that larger homes tend to report better process-related quality, though no major differences can be seen in terms of the structural variables. Larger nursing homes tend to be more privately owned, and have lower levels of self-rated health.

Associations of user questions with composite measure

As it was thought that there may be specific questions which may deviate from the overall composite score, we generated descriptive statisitics regarding the distribution of each included question by quantiles of the composite measure:

	1	2	3	4	5
N	359	359	358	359	359
sat range	-3.93 - -0.81	-0.81 - -0.2	-0.2 - 0.33	0.33 - 0.85	0.85 - 2.4
user5	63.650	71.457	74.947	79.219	85.235
user6	50.122	60.369	65.655	72.261	81.358
user7	51.862	59.624	66.537	71.463	79.727
user8	64.773	72.396	76.582	81.451	87.346
user9	55.997	65.791	70.827	76.356	84.146
user10	59.020	69.346	74.326	79.878	86.991
user11	35.707	43.344	49.424	57.332	66.799
user12	46.161	56.451	62.471	69.260	77.801
user13	88.037	93.404	95.409	97.135	98.369
user14	66.381	77.069	80.708	85.899	90.991
user16	79.274	86.249	89.732	93.056	96.474
user17	76.234	84.537	88.380	91.986	95.784
user18	47.654	55.516	63.390	69.946	78.335
user19	42.770	51.781	58.211	65.161	75.736
user21	66.289	73.910	77.693	80.809	88.435
user22	46.287	52.536	58.114	61.979	72.068
user23	74.320	82.155	86.107	89.730	94.491
user24	69.944	78.929	84.420	89.014	93.983
user25	38.368	43.570	46.632	50.624	56.499
user27	76.997	85.647	88.964	92.692	96.069

Sub-group analysis - Questionnaire completion

Due to the high rate of missingness for question 26 ("Who completed the questionnaire?"), we could not include this quite interesting data in our main analysis. The NBHW reported data for this question only for nursing home units with more than 7 responses, and as such, only 16% of nursing homes had data for this variable. This is far from sufficient to base reliable inference on, and we know that this missingness is associated with a factor (size) which is associated with resident satisfaction. Nonetheless, a quick look at the distribution of these data may be enlightening. Overall among nuring homes reporting data, 14.4% of questionnaires were filled out by the residents themselves, 22.5% had assistance filling out the survey, and 63.2% of questionnaires were filled out by somebody other than the user themselves. This high proportion of questionnaires completed by third parties is disquieting.

In a simple bivariate model, completion of the user survey by the resident themselves (with or without help) is associated with higher satisfaction scores (standardized beta coefficient of 0.12, 95% CI 0.01 - 0.22). Unfortunately, the NBHW does not report data on who the third party completing the questionnaire is - It is plausible that relatives completing the questionnaire are harsher in their judgements than the residents alone would be. Let's have a look as what predictors are associated with the resident themselves filling out the questionnaire with or without help:

	Effect	Lower 0.95	Upper 0.95
residentcouncil	0.157	0.000	0.315
actionplan	0.053	-0.037	0.143
meals	-0.005	-0.164	0.154
safetyroutines	0.062	-0.096	0.220
carecoord	0.017	-0.236	0.269
medreview	-0.117	-0.351	0.116
activity	0.132	-0.006	0.271
private	-0.043	-0.135	0.049
size	-0.402	-2.481	1.677
rns	-0.001	-0.002	0.001
staff	-0.010	-0.015	-0.005
edu	-1.135	-2.875	0.606
typegen	0.234	0.113	0.355
typedem	-0.112	-0.217	-0.007
typeserv	0.194	0.118	0.269
srhtot	0.298	0.098	0.497
userresponse	0.038	0.016	0.059

Given the low sample size, we see few robust effects. Among our process and structure variables, resident councils and activities appeared to have a positive association with self-completion, while staffing ratio appears to have a weak association with the percentage of questionnaires filled out by the resident - homes with higher staffing ratios to a greater extent are associated with surveys being completed by third parties. Among control variables, General care and short-term facilities have higher rates of resident completed surveys, while dementia facilities have lower rates. As may be expected, self rated health was positively correlated with self-completion of the survey, as was the response rate to they survey itself.

Alternate models

As in any secondary analysis of data, a number of decisions have been made in executing the analysis. Here, we present a set of analyses to explore possible alternative choices and evaluate the sensitivity of our findings to alternate methodologies. In addition to the additional analyses we present here, we also provide an interactive tool with which readers may specify alternate sets of weights for each question in the user survey, allowing permitting the exploration of the sensitivity of our findigngs to alteranate outcome specifictations.

Kajonius & Kazemi (2016) chose for instance to analyze the user survey using question 24 (relating to the users overall satisfaction with nursing home care) as the dependant variable. Lets go ahead and take a look at what our results would look like if we had taken that route. (Note that there are problems with the distribution of residuals with this approach given the skewed distribution of individual questions, with a significant portion of nursing homes having 100% of their residents report positive overall satisfaction)

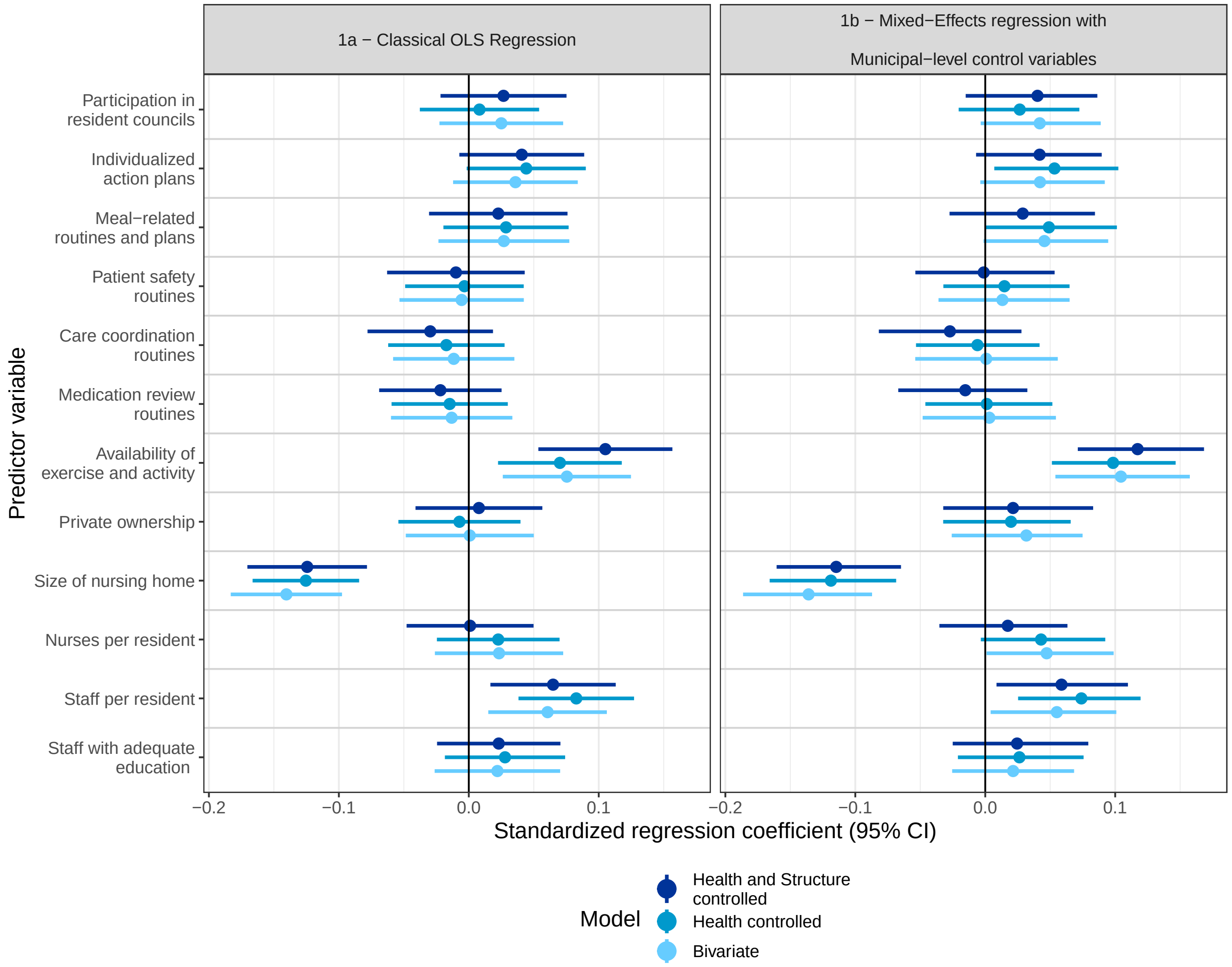

We see that using only question 24 as the outcome, the results are quite a bit weaker. In this reading, only physical activity, the size of the nursing home, and the ratio of staff per resident are significant at the $p < 0.05$ level.

We can also check to see if the larger number of small homes might be "washing out" effects relevant to a larger number of individual patients by weighting our data by the size of the nursing home. Note that we report only the OLS model, as lme4 had some difficulty generating appropriate confidence intervals for weighted observations. We welcome more talented programmers than ourselves to pursue this issue.

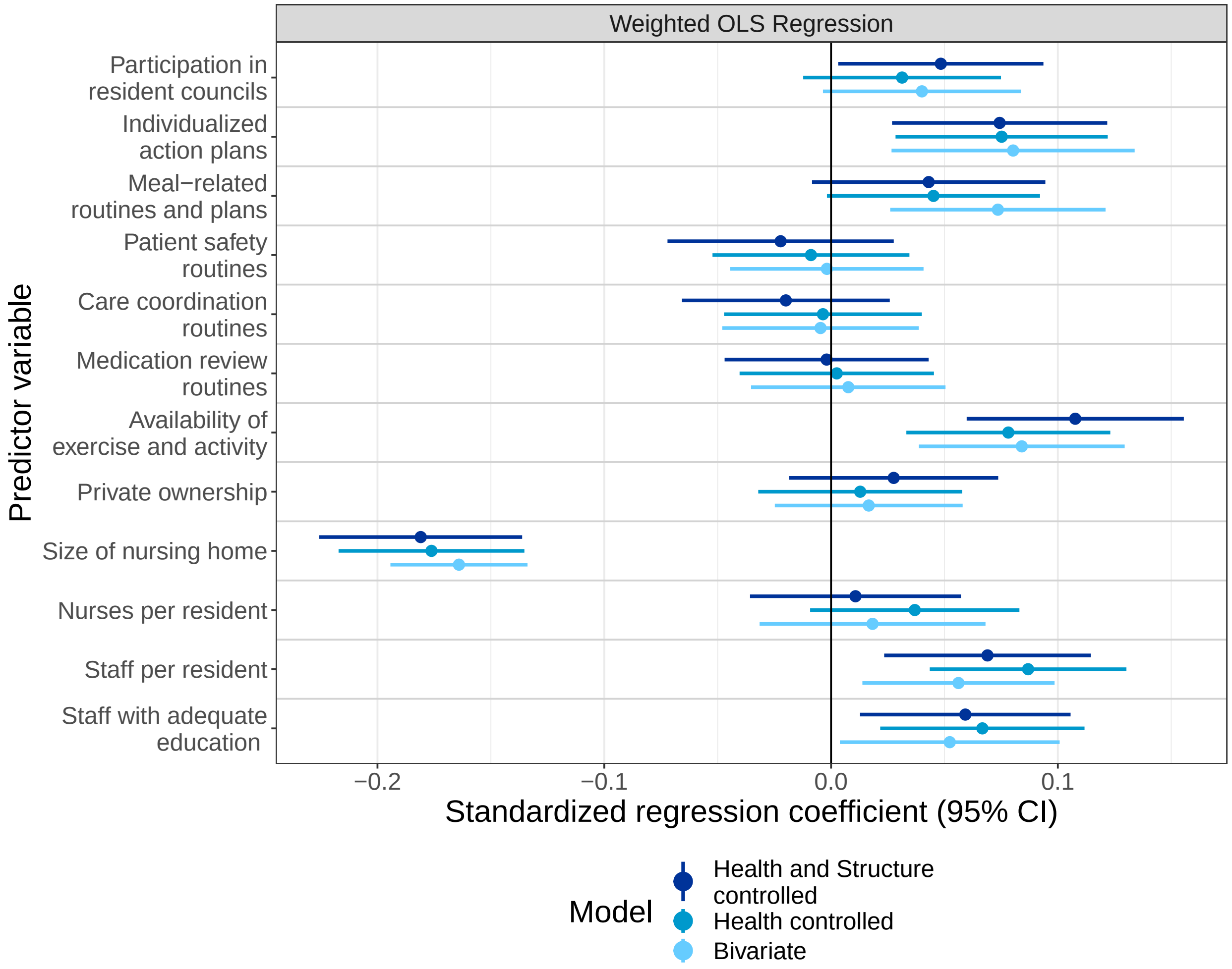

We find similar results as compared with our standard OLS regression model.

While we chose to apply a random effects model to control for municipal level effects, another alternative would be to include an indicator for municipaly as a fixed effect. Doing so produces the following results:

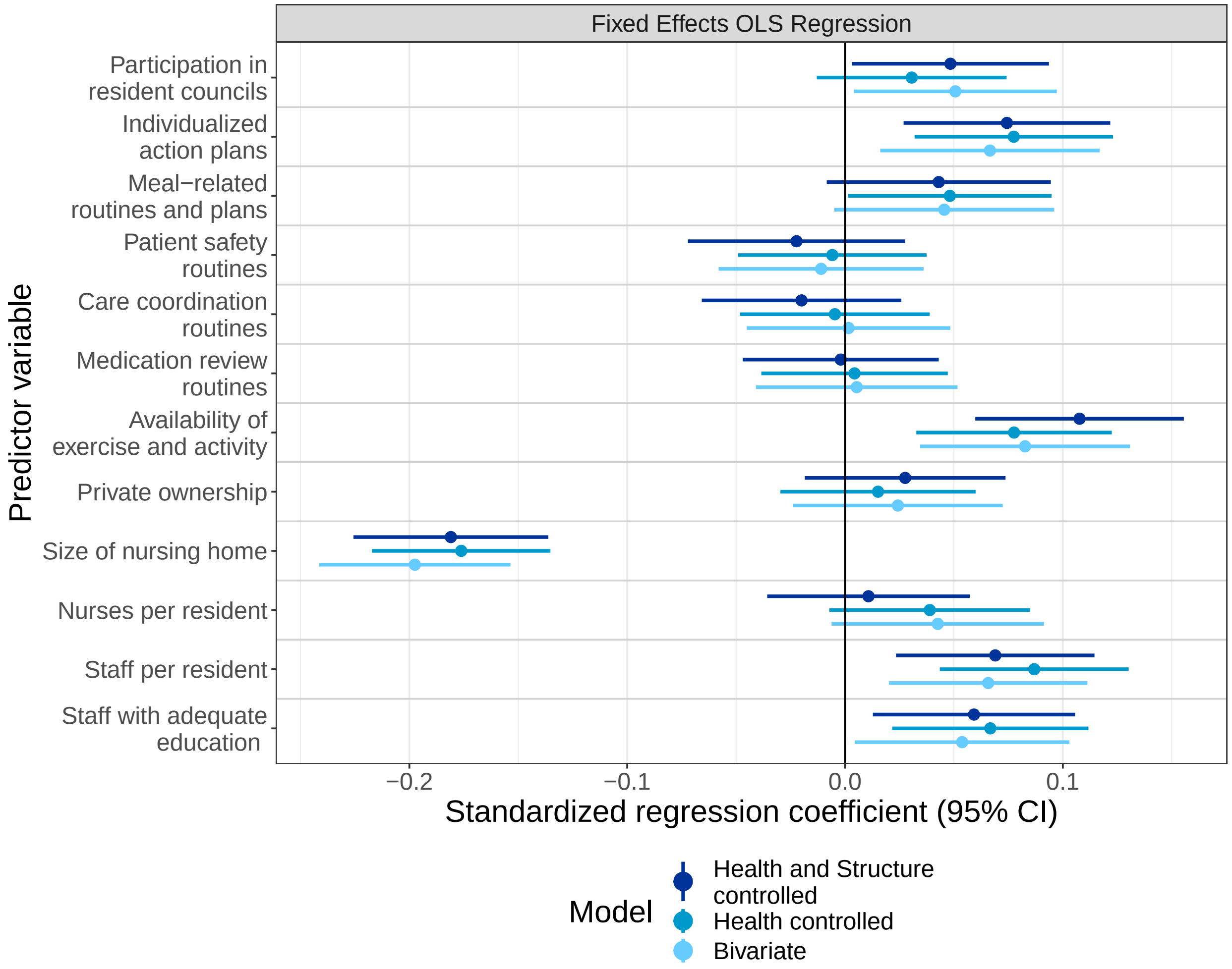
Fixed Effects OLS Regression
Predictor variable
Participation in resident councils
Individualized action plans
Meal−related routines and plans
Patient safety routines
Care coordination routines
Medication review routines
Availability of exercise and activity
Private ownership
Size of nursing home
Nurses per resident
Staff per resident
Staff with adequate education
−0.2
−0.1
0.0
0.1
Standardized regression coefficient (95% CI)
Model
Health and Structure controlled
Health controlled
Bivariate
