# Supplementary material for: Small is beautiful? Explaining resident satisfaction in Swedish nursing home care
Source: BMC Health Serv Res. 2019 Nov 25;19:886. doi: 10.1186/s12913-019-4694-9 (PMC6878673; doi:10.1186/s12913-019-4694-9)
Supplement: Supplementary file 2 — Additional file 2. Survey_questions. This document details the specific questions from the two NHBW surveys constituting the aggregate variables included as independent variables in the regression analysis reported in this manuscript. [file 12913_2019_4694_MOESM2_ESM.docx]

**Additional file 2 – Survey question translations and aggregations**

| **Survey – Question number** | **Survey Questionnaire item** | **Model variable** |
| --- | --- | --- |
| User - 1 | How do you judge your general state of health?  [Hur bedömer du ditt allmänna hälsotillstånd?] | Aggregate Self-Rated Health |
| User -2 | Are you troubled by worry, fear or anxiety?  [Har du besvär av ängslan, oro eller ångest?] |  |
| User - 3 | How is your mobility indoors?  [Hur är din rörlighet inomhus?] |  |
| User - 20 | Are you troubled by loneliness?  [Händer det att du besväras av ensamhet?] |  |
| User - 4 | Did you get placed at the nursing home you wanted?  [Fick du plats på det äldreboende du ville bo på?] | Excluded |
| User - 5 | Are you happy with your room or apartment?  [Trivs du med ditt rum eller lägenhet?] | Aggregate resident satisfaction |
| User – 6 | Are the common areas pleasant?  [Är det trivsamt i de gemensamma utrymmena?] |  |
| User – 7 | Is the outdoor area around your nursing home pleasant?  [Är det trivsamt utomhus runt ditt boende?] |  |
| User – 8 | How does the food usually taste?  [Hur brukar maten smaka?] |  |
| User – 9 | Do you feel that mealtimes at your nursing home are pleasant?  [Upplever du att måltiderna på ditt äldreboende är en trevlig stund på dagen?] |  |
| User – 10 | Does the staff at your nursing home usually have time to do their work with you?  [Brukar personalen ha tillräckligt med tid för att kunna utföra sitt arbete hos dig?] |  |
| User – 11 | Does the staff at your nursing home usually tell you about temporary changes beforehand?  [Brukar personalen meddela dig i förväg om tillfälliga förändringar?] |  |
| User – 12 | Can you usually affect what times you get assistance?  [Brukar du kunna påverka vid vilka tider du får hjälp?] |  |
| User – 13 | Does the staff usually treat you well?  [Brukar personalen bemöta dig på ett bra sätt?] |  |
| User – 14 | Does the staff usually take your opinion and wishes into account when deciding on how assistance should be provided?  [Brukar personalen ta hänsyn till dina åsikter och önskemål om hur hjälpen ska utföras?] |  |
| User – 16 | How safe or unsafe does your nursing home feel?  [Hur tryggt eller otryggt känns det att bo på ditt äldreboende?] |  |
| User – 17 | Do you feel confidence in the staff at your nursing home?  [Känner du förtroende för personalen på ditt äldreboende?] |  |
| User – 18 | How satisfied or dissatisfied are you with the activities offered at your nursing home?  [Hur nöjd eller missnöjd är du med de aktiviteter som erbjuds på ditt äldreboende?] |  |
| User – 19 | Are the opportunities to go outside good or bad?  [Är möjligheterna att komma utomhus bra eller dåliga?] |  |
| User – 21 | How easy or hard is it to contact a nurse if you need one?  [Hur lätt eller svårt är det att få träffa sjuksköterska vid behov?] |  |
| User – 22 | How easy or hard is it to contact a doctor if you need one?  [Hur lätt eller svårt är det att få träffa läkare vid behov?] |  |
| User – 23 | How easy or hard is it to contact the staff at your nursing home if you need them?  [Hur lätt eller svårt är det att få kontakt med personalen på ditt äldreboende, vid behov?] |  |
| User – 24 | How satisfied or dissatisfied are you with your nursing home overall?  [Hur nöjd eller missnöjd är du sammantaget med ditt äldreboende?] |  |
| User – 25 | Do you know where to turn if you want to give your opinion or complaints about the nursing home?  [Vet du vart du ska vända dig om du vill framföra synpunkter eller klagomål på äldreboendet?] |  |
| User – 27 | How do you think the cooperation between you and the nursing home works?  [Hur tycker du att samarbetet mellan dig och äldreboendet fungerar?] |  |
| User – 26 | Who answered/filled in the questionnaire?  [Vem besvarade/fyllde i frågeformuläret?] | Excluded |
| Unit – 1 | The unit offers each resident the opportunity to participate in resident councils.  [Enheten erbjuder varje person möjlighet att delta i borådsmöten.] | Participation in resident councils |
| Unit – 1a | The unit offers each resident the opportunity to participate in resident councils, per time period  [Enheten erbjuder varje person möjlighet att delta i borådsmöten, enligt intervall]. |  |
| Unit – 2 | Portion of residents with a current action plan  [Andel personer med aktuell genomförandeplan]. Procent. | Individualized action plans |
| Unit – 3 | Portion of residents with an action plan which contains documentation about how the resident participated in the creation of the action plan  [Andel personer med en plan som innehåller dokumentation om hur personen deltagit vid upprättande/förändring av sin genomförandeplan. Procent.] |  |
| Unit – 4 | A current routine exists for the execution of all meals of the day  [Aktuell rutin finns för genomförande av dygnets alla måltider.] | Meal-related routines and plans |
| Unit – 5 | Portion of residents whose current action plan contains information about the resident’s desires and needs in connection with meals  [Andel personer med aktuell genomförandeplan som innehåller information om den äldres önskemål och behov i samband med måltiderna. Procent.] |  |
| Unit – 6a | A current routine for the suspicion/discovery of that the resident has been subjected to violence or assault by a relative  [Aktuell rutin för misstanke/upptäckt av att den enskilde utsatts för våld eller övergrepp av en anhörig/närstående.] | Patient safety routines |
| Unit – 6b | A current routine exists for the suspicion/discovery of that the resident is addicted to medications  [Aktuell rutin för misstanke/upptäckt av att den enskilde är beroende av/missbrukar läkemedel.] |  |
| Unit – 6c | A current routine exists for the suspicion/discovery of that the resident is addicted to alcohol or other addictive substances (not medication)  [Aktuell rutin för misstanke/upptäckt av att den enskilde är beroende av/missbrukar alkohol eller andra beroendeframkallande medel (ej läkemedel).] |  |
| Unit – 7 | A current routine exists for how the staff should cooperate with relatives of those receiving interventions at the nursing home.  [Aktuell rutin för hur personalen samarbetar med anhöriga till personer som får insatser vid enheten.] | Excluded |
| Unit – 8 | There is access to a gym  [Det finns tillgång till träningslokal.] | Excluded |
| Unit – 8a | The nursing home offers access to activities  [Enheten erbjuder tillgång till aktiviteter.] | Availability of exercise and activities |
| Unit – 8b | Residents of the nursing home have access to strength and balance exercise  [Personer vid enheten har tillgång till styrke- och balansträning.] |  |
| Unit – 9 | Current routines for planning the residents medical care in cooperation with various actors exist. It is documented how the resident has been involved in the planning of medical care.  [Aktuella rutiner finns för hur planering av den enskildes hälso- och sjukvård (vårdplanering) genomföras i samverkan med olika aktörer. Det är dokumenterat i journal hur den enskilde varit delaktig vid vårdplaneringen.] | Care Coordination routines |
| Unit – 10 | Current routines for planning the residents medical care in cooperation with the resident, the resident’s doctor, and municipal healthcare staff.  [Aktuella rutiner finns för hur planering av den enskildes hälso- och sjukvård (vårdplanering) ska genomföras i samverkan med den enskilde och ansvarig läkare och kommunal hälso- och sjukvårdspersonal samt omsorgspersonal.] |  |
| Unit – 11 | Current routines for how thorough medication reviews should be performed exist, and the resident’s participation is documented in the journal.  [Aktuella rutiner finns för hur fördjupade läkemedelsgenomgångar ska genomföras, den enskildes delaktighet är dokumenterad i journalen.] | Medication Review routines |
| Unit – 12 | Current routines for how thorough medication reviews should be performed in cooperation with the resident, the residents doctor, the responsible nurse and care staff exist.  [Aktuella rutiner finns för hur fördjupade läkemedelsgenomgångar ska genomföras i samverkan med den enskilde och läkare samt med ansvarig sjuksköterska och omsorgspersonal.] |  |
| Unit – 13 | Number of nurses per resident plus any home care patients, weekdays  [Antal sjuksköterskor per antalet bostäder plus eventuella hemsjukvårdspatienter, vardagar. Procent.] | Nurses per resident |
| Unit – 14 | Number of nurses per resident plus any home care patients, weekends/holidays  [Antal sjuksköterskor per antalet bostäder plus eventuella hemsjukvårdspatienter, helgdagar. Procent]. |  |
| Unit – 15 | Number of care staff per resident, weekdays  [Antal omsorgspersonal per antalet bostäder vid enheten, vardagar. Procent.] | Staff per resident |
| Unit – 16 | Number of care staff per resident, weekends/holidays  [Antal omsorgspersonal per antalet bostäder vid enheten, helgdagar. Procent.] |  |
| Unit – 17 | Portion of staff with adequate education, weekdays  [Andel omsorgspersonal med adekvat utbildning, vardagar. Procent.] | Staff with adequate education |
| Unit – 18 | Portion of staff with adequate education, weekends/holidays  [Andel omsorgspersonal med adekvat utbildning, helgdagar. Procent.] |  |
